# Comprehensive investigation of isotherm, RSM, and ANN modeling of CO_2_ capture by multi-walled carbon nanotube

**DOI:** 10.1038/s41598-024-55836-6

**Published:** 2024-03-01

**Authors:** Zohreh Khoshraftar, Ahad Ghaemi, Alireza Hemmati

**Affiliations:** School of Chemical, Petroleum and Gas Engineering, Iran University of Science and Technology, P.O. Box 16765-163, Tehran, Iran

**Keywords:** Multi-walled carbon nanotube, Fe–Ni/AC catalyst, CVD, CO_2_ adsorption, RSM, ANN, Environmental chemistry, Chemical engineering

## Abstract

Chemical vapor deposition was used to produce multi-walled carbon nanotubes (MWCNTs), which were modified by Fe–Ni/AC catalysts to enhance CO_2_ adsorption. In this study, a new realm of possibilities and potential advancements in CO_2_ capture technology is unveiled through the unique combination of cutting-edge modeling techniques and utilization of the recently synthesized Fe–Ni/AC catalyst adsorbent. SEM, BET, and FTIR were used to analyze their structure and morphology. The surface area of MWCNT was found to be 240 m^2^/g, but after modification, it was reduced to 11 m^2^/g. The modified MWCNT showed increased adsorption capacity with higher pressure and lower temperature, due to the introduction of new adsorption sites and favorable interactions at lower temperatures. At 25 °C and 10 bar, it reached a maximum adsorption capacity of 424.08 mg/g. The optimal values of the pressure, time, and temperature parameters were achieved at 7 bar, 2646 S and 313 K. The Freundlich and Hill models had the highest correlation with the experimental data. The Second-Order and Fractional Order kinetic models fit the adsorption results well. The adsorption process was found to be exothermic and spontaneous. The modified MWCNT has the potential for efficient gas adsorption in fields like gas storage or separation. The regenerated M-MWCNT adsorbent demonstrated the ability to be reused multiple times for the CO_2_ adsorption process, as evidenced by the study. In this study, a feed-forward MLP artificial neural network model was created using a back-propagation training approach to predict CO_2_ adsorption. The most suitable and efficient MLP network structure, selected for optimization, consisted of two hidden layers with 25 and 10 neurons, respectively. This network was trained using the Levenberg–Marquardt backpropagation algorithm. An MLP artificial neural network model was created, with a minimum MSE performance of 0.0004247 and an R^2^ value of 0.99904, indicating its accuracy. The experiment also utilized the blank spreadsheet design within the framework of response surface methodology to predict CO_2_ adsorption. The proximity between the Predicted R^2^ value of 0.8899 and the Adjusted R^2^ value of 0.9016, with a difference of less than 0.2, indicates a high level of similarity. This suggests that the model is exceptionally reliable in its ability to predict future observations, highlighting its robustness.

## Introduction

**Figure 1:**
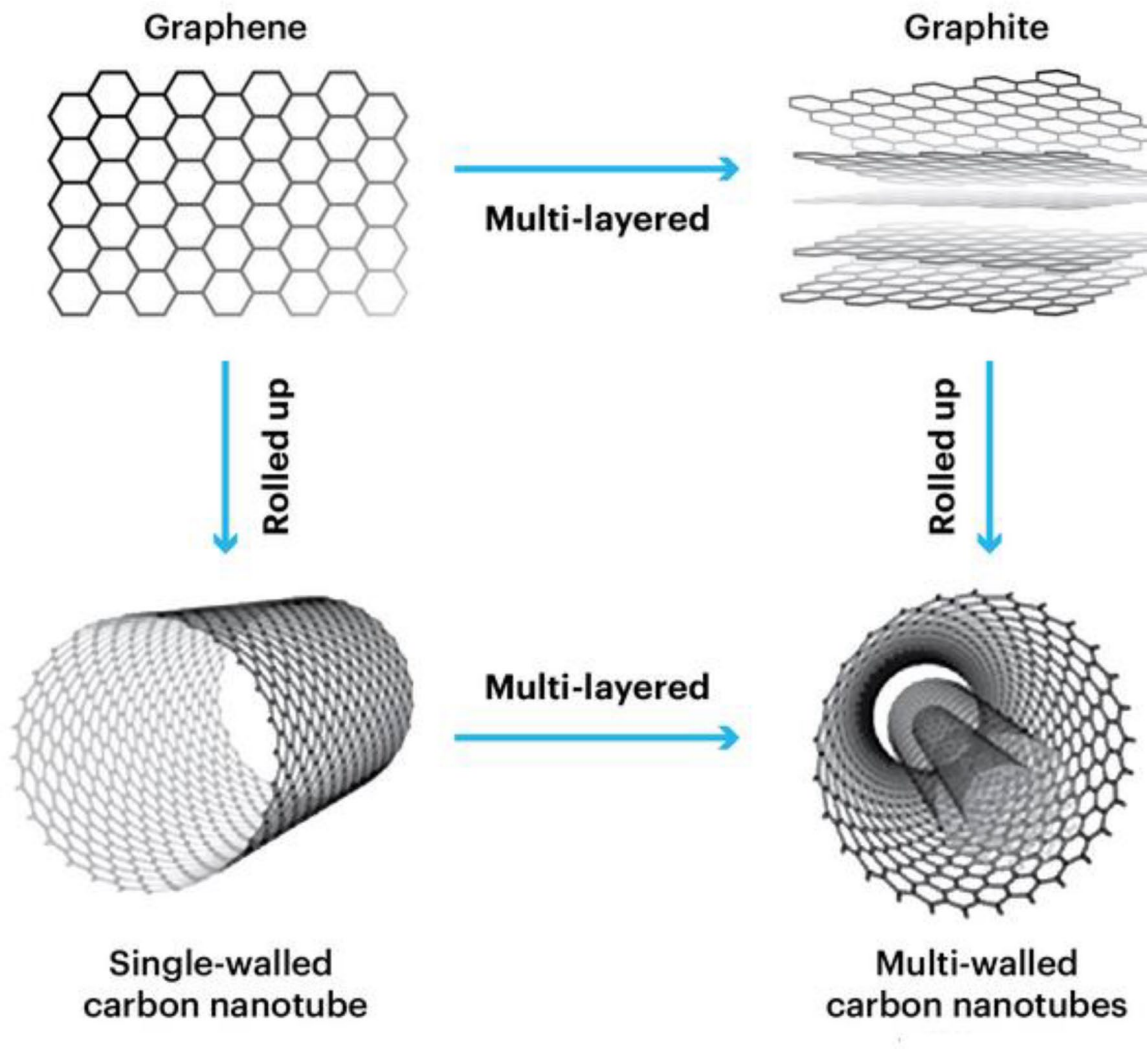
Structure of SWCNTs and MWCNTs^49^.

Rising sea levels, extreme weather, declining biodiversity, water scarcity, and melting of polar ice. Unfortunately, these terms are becoming more common as we go due to the acceleration of climate change contributed by emissions of greenhouse gases such as CO_2_ and methane (CH_4_). For global warming to conform to the original limit, the Intergovernmental Panel on Climate Change (IPCC) report in 2022 claimed that the worldwide greenhouse gas emissions need to peak by 2025 and be lessened by 43% by 2030^1–3^. Adsorption is recognized as one of the primary techniques for capturing CO_2_^4^, along with chemical absorption^5–7^, membrane separation, and cryogenic distillation^1^. Although chemical absorption currently receives more attention and usage in research and implementation^8^, adsorption is gaining significant recognition as an effective CO_2_ separation technology. This is attributed to its notable CO_2_ adsorption capacity and selectivity, as well as its low energy requirement for regeneration^9^. Additionally, most adsorbents used in adsorption processes possess advantageous characteristics such as wide availability, affordability, and thermal stability. These factors contribute to the appeal and potential of adsorption as a promising approach to mitigate CO_2_ emissions^10,11^. Pressure Swing Adsorption (PSA) technology is a popular and environmentally friendly solution widely adopted in modern society. It serves as a cost-effective and energy-efficient gas separation technique^12,13^. The utilization of cycling adsorption, specifically pressure swing adsorption (PSA) and temperature swing adsorption (TSA) methods for capturing CO_2_, has garnered significant interest. These techniques offer efficient means of separating and purifying CO_2_ from gas mixtures. In the case of PSA, the pressure is altered to achieve highly selective adsorption of CO_2_, effectively separating it from nitrogen (N_2_) and other components in the gas mixture^14^. While considerable progress has been made in the discovery of advanced materials for CO_2_ capture, there are still obstacles to overcome in terms of enhancing kinetics, capacity, selectivity, stability, and overall cost-effectiveness^15^. Porous materials, including zeolites^16–24^, metal–organic framework materials (MOFs)^25–27^, porous organic polymers (POPs)^28–30^, and porous carbon^31–35^, hold promise as solid adsorbents for CO_2_ capture. In recent times, there has been a surge of interest in the utilization of solid sorbents, particularly carbon nanotubes (CNTs), for the adsorption of CO_2_^36,37^. This heightened attention can be attributed to the remarkable properties exhibited by CNTs, including their exceptionally high surface area, unique hollow tubular structure, impressive mechanical strength, and outstanding thermal and chemical stability^38^. Moreover, CNTs possess the capability to efficiently adsorb CO_2_ and undergo regeneration at relatively low temperatures, resulting in significantly reduced energy consumption. These exceptional characteristics position CNTs as highly promising candidates for CO_2_ adsorption applications^39^. Differentiating from other carbon materials, carbon nanotubes (CNTs) are exceptional due to their one-dimensional structure. They can be described as elongated cylinders formed by rolling up graphene sheets with diameters at the nanoscale^40^. The cylindrical tubes have hemisphere fullerenes capping their ends. CNTs can be broadly categorized into two basic types based on the number of walls: single-walled carbon nanotubes (SWCNTs) and multi-walled carbon nanotubes (MWCNTs)^41^. Figure 1 illustrates that single-walled carbon nanotubes (SWCNTs) can be visualized as a single graphene sheet rolled into a seamless cylinder. On the other hand, multi-walled carbon nanotubes (MWCNTs) are composed of two or more concentric cylindrical shells of graphene sheets, arranged coaxially. These shells are held together by van der Waals forces between the adjacent layers, resulting in a central hollow core. This unique structure distinguishes SWCNTs from MWCNTs^42^. After conducting a review of the published work, it has been determined that the most effective method for synthesizing well-structured multi-walled carbon nanotubes (MWCNTs) is through chemical vapour deposition (CVD). The CVD approach offers several advantages, including the ability to control the geometry of the nanotubes, lower process temperatures, precise control over parameters, and reduced production costs^43,44^. In addition to chemical vapour deposition (CVD), other synthesis methods for multi-walled carbon nanotubes (MWCNTs) include^45–47^:

- *Arc Discharge Method*^48^ This method involves high-current arc discharges between two carbon electrodes in an inert atmosphere. The high temperatures generated in the arc vaporize a carbon electrode, leading to the formation of MWCNTs.
- *Laser Ablation Method* In this method, a high-powered laser is focused on a carbon target in a reaction chamber filled with an inert gas. The laser vaporizes the carbon target, and MWCNTs are formed as a result of the condensation of the vapour.
- *Catalytic Chemical Vapor Deposition (CCVD)*:Similar to CVD, CCVD involves the decomposition of carbon-containing precursors on a catalyst surface. The catalyst acts as a nucleation site for the growth of MWCNTs. Different catalysts and precursor gases can be used to control the properties of the nanotubes.
- *Floating Catalyst Method* This method involves the thermal decomposition of hydrocarbon gases in the presence of a catalyst. The catalyst particles are suspended in a reactor, allowing the MWCNTs to grow on the catalyst surface. The MWCNTs are then separated by various post-processing techniques.

Comparing these synthesis methods, CVD stands out as the most effective for synthesizing well-structured MWCNTs due to several advantages it offers. These advantages include better control over the geometry of nanotubes, lower process temperatures that minimize defects, precise control over growth parameters, and reduced production costs. However, the other methods also have their advantages and applications. For example, the arc discharge method and laser ablation method can produce high-quality MWCNTs in small quantities, while CCVD and the floating catalyst method are more scalable for mass production. The choice of synthesis method depends on specific requirements, such as desired nanotube properties, scalability, and cost-effectiveness^45^.

Several studies have investigated the CO_2_ sorption capacity of amine-functionalized multi-walled carbon nanotubes (MWCNTs), and the overall outcomes have been promising. These studies have consistently demonstrated that amine-functionalized MWCNTs exhibit a high affinity for CO_2_ adsorption. The amine functional groups on the surface of the nanotubes facilitate strong interactions with CO_2_ molecules, leading to enhanced sorption capacity. Additionally, the high surface area and porous structure of MWCNTs further contribute to the increased CO_2_ uptake. Overall, these selected studies have highlighted the potential of amine-functionalized MWCNTs as efficient sorbents for carbon capture and storage applications, offering a promising avenue for mitigating CO_2_ emissions^38,39,50–52^.

Response surface methodology (RSM) is a multivariate nonlinear modelling that is gaining popularity among researchers. It allows us to understand the impact of input variables on the response output and determine the significance of individual or combined independent variables in a process. RSM offers several advantages, including a shorter time requirement, fewer experiments, and the ability to predict responses accurately^53–56^. Artificial neural network (ANN) modelling has gained significant attention among researchers due to its ability to capture nonlinear relationships between independent variables and solve problems that cannot be addressed by traditional statistical methods. ANN provides a powerful tool for understanding complex patterns and predicting outcomes, making it a hot topic in research^57^.

Recent research has unveiled a highly efficient modified multi-walled carbon nanotube (M-MWCNT) as a promising adsorbent for carbon dioxide gas. To better understand the properties of the materials derived from MWCNT and M-MWCNT, several characterization techniques such as field emission scanning electron microscopy (FESEM), and Fourier-transform infrared spectroscopy (FTIR) were employed. These analyses provided valuable insights into the morphology and structure of the materials. Furthermore, a N_2_ adsorption–desorption study was conducted to evaluate the impact of the treatment process on the morphology of the materials. This study revealed valuable information about the adsorption capacity, kinetics, isotherms, and thermodynamics of the M-MWCNT adsorbent. This study presents a novel approach by utilizing modelling techniques to analyze various aspects of CO_2_ capture, including isotherm, kinetics, thermodynamics, response surface methodology (RSM) and artificial neural networks (ANN) modelling. The RSM and ANN modelling techniques further enhance the understanding and optimization of the adsorption process. The application of these modelling techniques in the context of CO_2_ capture is a significant contribution to the field. Moreover, this study introduces a unique synthesis method for the development of an adsorbent specifically designed for CO_2_ capture. This marks the first time that such a synthesized adsorbent has been applied for CO_2_ capture purposes. The novelty lies in the combination of the advanced modelling techniques and the use of this newly synthesized adsorbent by Fe–Ni/AC catalyst, which opens up new possibilities and potential advancements in CO_2_ capture technology. In the field of CO_2_ capture, a regeneration process that has proven to be especially effective involves a series of repeated adsorption and desorption cycles, typically performed around 10 times.

## Materials and methods

### Materials

The necessary chemicals for the experiment were sourced from reputable suppliers. Hydrochloric acid (HCl 32%), dichloromethane (CH_2_Cl_2_ 99%), and nitric acid (HNO_3_ 55%) were provided by Mojallali Company located in Tehran, Iran. These chemicals were chosen for their high purity and reliable quality, ensuring accurate and consistent results in the experiment. To create the desired atmosphere for the experiment, gases were purchased from Arman Gas Company. Carbon dioxide (CO_2_ 99.99%), argon (Ar 99.99%), acetylene (C_2_H_2_ 99.99%), and nitrogen (N_2_ 99.99%) were acquired. These gases were carefully selected to meet the requirements of the experiment, guaranteeing reliable and consistent gas compositions for accurate and precise measurements.

### Synthesis method

To create the Fe–Ni/AC catalyst, a 200 mL conical flask was used. Inside the flask, 5 g of pure Jacobi activated carbon (AC) was added. Then, 50 mL of distilled water was poured into the flask. Additionally, Fe (NO_3_)_3_.9H_2_O and Ni (NO_3_)_2_.6H_2_O, both with a concentration of 0.25M, were included in the mixture. Before subjecting the slurry to a drying process in an oven set at a temperature of 373 K for a duration of 12 h, it was necessary to stir the mixture. The stirring process lasted for 20 min. This step was taken to ensure proper mixing and distribution of the components within the slurry, which would subsequently contribute to the desired properties and performance of the Fe–Ni/AC catalyst. After the slurry was cooled down to room temperature, the material was crushed before being sieved using a 200 µm sieve. This crushing and sieving process was employed to obtain particles of a desired size range, which would improve the effectiveness of the catalyst. Once the catalyst particles were obtained, they were subjected to a heating process at a temperature of 673 K for 6 h. The purpose of this heating step was to eliminate any remaining nitrates and humidity present in the catalyst. Removing nitrates is important as they can act as potential contaminants during catalytic reactions, while humidity can affect the stability and performance of the catalyst. By heating the catalyst, these impurities were effectively eliminated, resulting in a catalyst with improved purity and stability. To begin the process, the 5 g of obtained catalyst was carefully placed into a ceramic boat. This boat was then positioned inside the quartz tube, which had a diameter of 90 mm. The entire setup was placed within a CVD (Chemical Vapor Deposition) horizontal tube furnace. To create a controlled environment, a flow of argon gas was introduced over the Fe–Ni/AC catalyst at a rate of 30 mL/min. This flow of argon served to purge the air present within the system, ensuring that any potential contaminants or unwanted gases were removed. To gradually increase the temperature within the system, a heating rate of 10 °C per minute was employed. This allowed for a controlled and uniform heating process, ensuring that the catalyst reached the desired temperature without any sudden fluctuations. Overall, this setup and procedure in the CVD horizontal tube furnace allowed for the activation and preparation of the Fe–Ni/AC catalyst under controlled conditions, ensuring its stability and effectiveness in subsequent chemical vapour deposition processes. During the experiment, at a temperature of 973 K, the flow rate of argon gas was adjusted to 250 mL/min. This flow rate was chosen to provide sufficient gas circulation within the reactor, ensuring adequate heat transfer and distribution. Simultaneously, acetylene gas was introduced into the system at a flow rate of 100 mL/min. This specific flow rate was selected based on the desired reaction conditions and the requirements of the experiment. The acetylene gas was allowed to flow for a duration of 30 min, enabling the reaction to take place and the desired chemical transformations to occur. After completing the reaction, the furnace was cooled down to room temperature. At this stage, the flow of acetylene gas was ceased, and the reactor was purged. The incorporation of acetylene gas during the activation of Fe–Ni/AC (Iron-Nickel on Activated Carbon) catalysts is commonly performed to boost their catalytic effectiveness. Acetylene serves various purposes in this activation process:

- *Surface Purification* Acetylene aids in eliminating impurities and contaminants from the catalyst surface, ensuring a clean and active surface for catalytic reactions.
- *Carburization* As a hydrocarbon, acetylene can undergo carburization reactions during activation, introducing carbon into the catalyst structure. This alters the surface properties of the catalyst, enhancing its catalytic activity.
- *Reduction* Acetylene participates in reduction reactions, reducing metal oxides in the catalyst. This reduction step is crucial for activating metal catalysts, converting metal oxides into their active metallic form.
- *Formation of Active Sites* The introduction of acetylene generates active sites on the catalyst surface, crucial for facilitating the desired catalytic reactions.
- *Improved Catalytic Performance* Overall, the addition of acetylene enhances the catalytic performance of Fe–Ni/AC catalysts, leading to increased efficiency and selectivity in catalyzed reactions.

The purging process involved introducing a flow of inert gas, such as argon, into the reactor at a rate of 20 mL/min. This step was performed to remove any remaining traces of acetylene gas from the reactor, ensuring the safety and integrity of subsequent processes or analyses.

**Figure 2:**
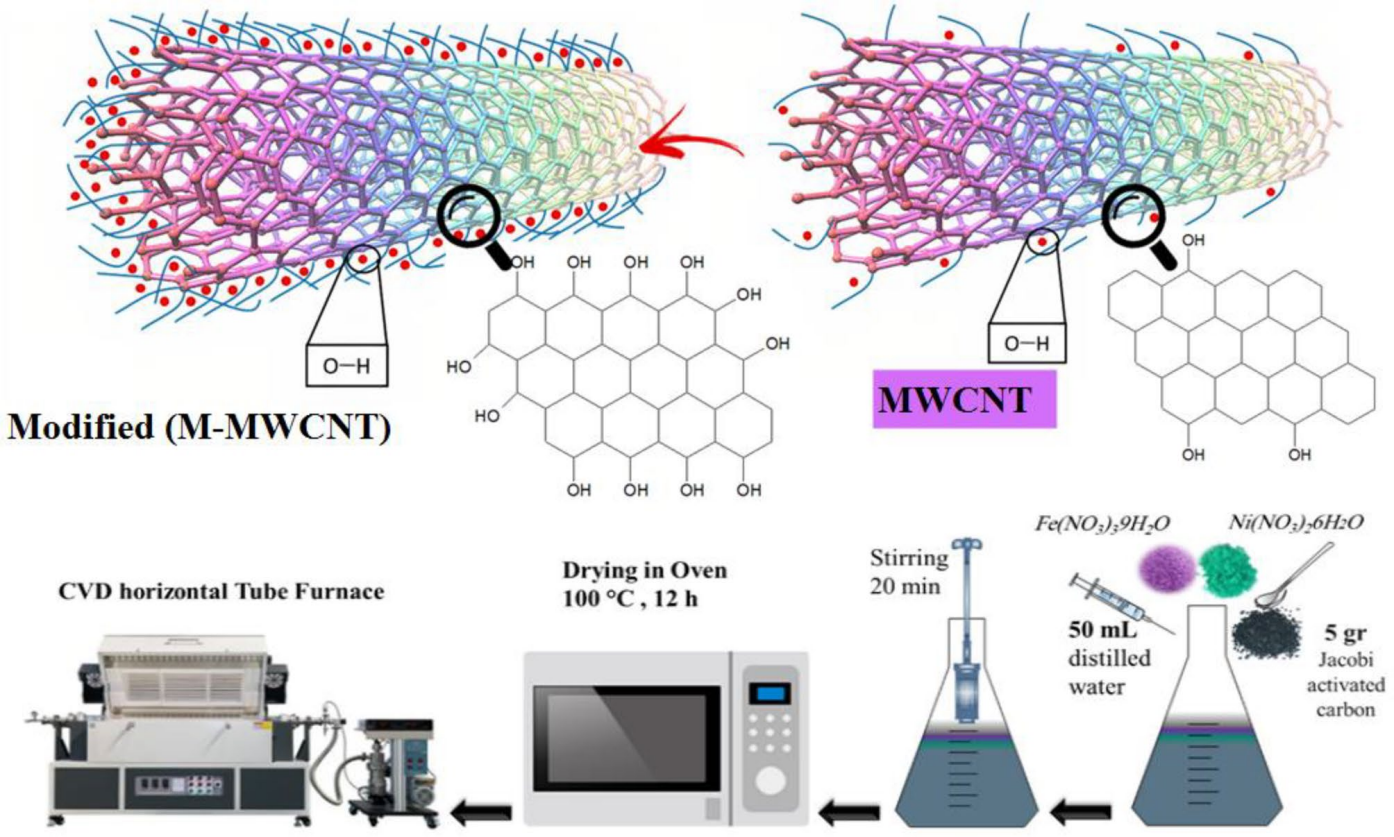
The steps of preparation of M-MWCNT.

The synthesis of synthesized MWCNT was conducted by the methodology outlined in a previous study^45^. A dispersion of synthesized multi-walled carbon nanotubes (MWCNT) was created by combining 0.5 g of MWCNT with 100 ml of dichloromethane. To facilitate the dispersion, the mixture was subjected to sonication for 0.5 h at a temperature of 328 K. Following the sonication step, the resulting solution was stirred for 5 min. This stirring process aided in ensuring proper mixing of the MWCNT with the solvent. To further modify the MWCNT, a mixture of 30 ml of hydrochloric acid and 30 ml of nitric acid was added to the solution. The resulting mixture was then stirred for an hr at a temperature of 333 K. This stirring and heating process facilitated the evaporation of the residual dichloromethane present in the solution. In summary, to modify the MWCNT, they were dispersed in dichloromethane through sonication, followed by stirring. Subsequently, hydrochloric acid and nitric acid were added, and the resulting mixture was stirred and heated to remove any remaining dichloromethane. After the crushing and sieving process, the catalyst sample was further treated. A mixture of 1g of NaOH and 100 ml of deionized water was applied to the sample. This was done to adjust the pH of the solution to approximately 7. Maintaining a neutral pH is essential as it helps to optimize the catalytic activity of the Fe–Ni/AC catalyst. Once the pH was adjusted, the catalyst sample was dried in an oven set at a temperature of 353 K for 24 h. The purpose of this drying process was to remove any remaining moisture from the catalyst, ensuring its stability and preventing potential chemical reactions or degradation during storage or subsequent use. By effectively drying the catalyst, its performance and longevity can be enhanced. Figure 2 illustrates the sequential steps involved in the preparation of the synthesis for the desired product.

### Adsorption setup

**Figure 3:**
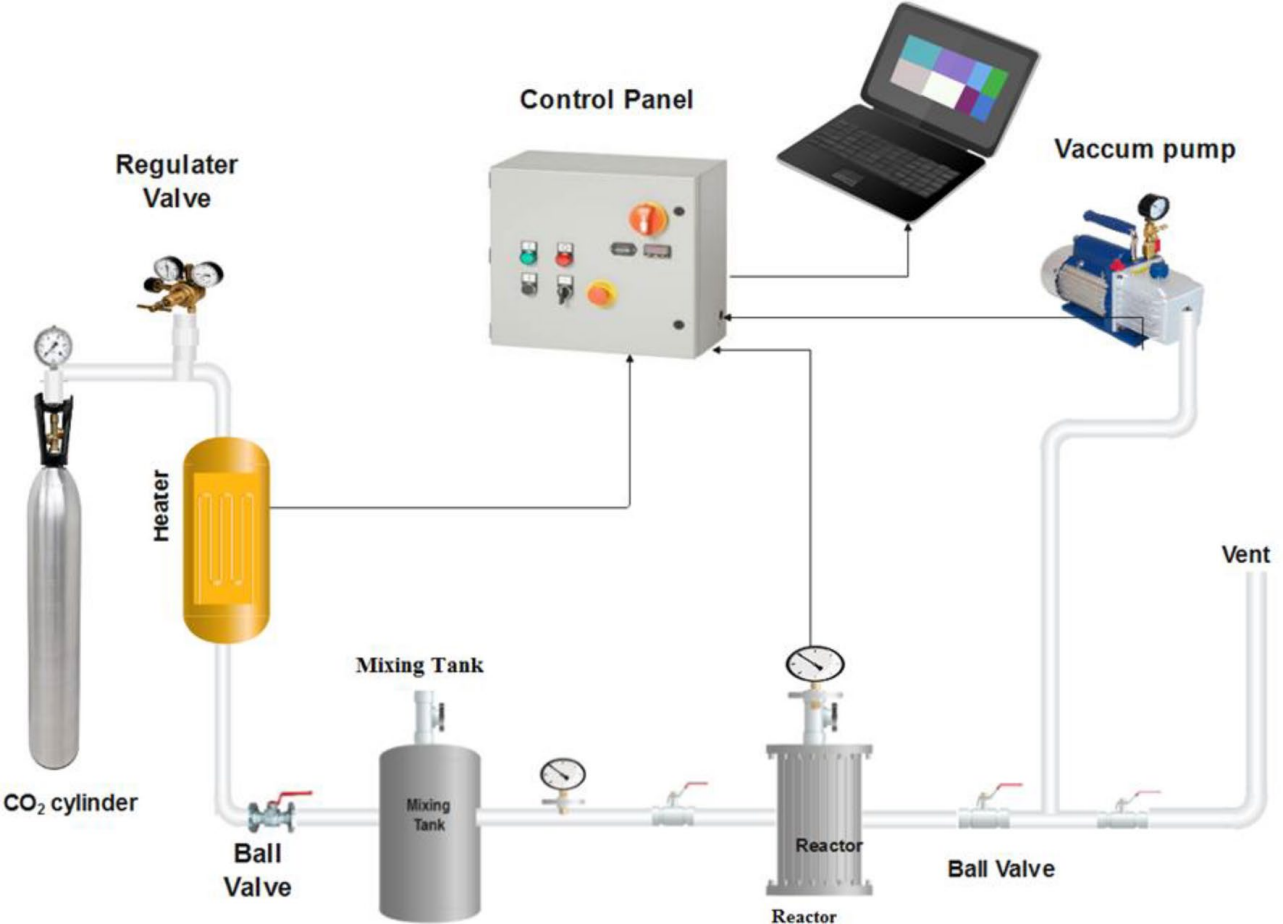
Setup of CO_2_ adsorption.

The laboratory adsorption setup was used to perform CO_2_ adsorption using M-MWCNT. A schematic of the setup is shown in Fig. 3. The setup can be divided into three main parts for better understanding^33^:

(I) Gas Injection System: This part of the setup is responsible for injecting the CO_2_ gas into the reactor. It ensures a controlled and precise flow of CO_2_ gas into the system. (II) CO_2_ Reactor System: This part of the setup is where the actual adsorption process takes place. The M-MWCNT material interacts with the CO_2_ gas, leading to its adsorption. The reactor system is designed to optimize the adsorption process and enhance the efficiency of CO_2_ removal. (III) Analysis of CO_2_ Pressure: After the adsorption process, it is crucial to measure and analyze the CO_2_ pressure in the reactor. This step helps in evaluating the effectiveness of the adsorption process and determining the amount of CO_2_ removed by the M-MWCNT material. By dividing the laboratory setup into these three parts, it becomes easier to understand the different functions and processes involved in the CO_2_ adsorption experiment. To prepare the adsorbent for dehumidification and pre-adsorption of CO_2_, a heating process was employed using nitrogen gas at a temperature of 388 K. The nitrogen gas was heated for 30 min to ensure proper activation and removal of any residual moisture.

Following the heating process, the system was then subjected to a vacuum for 40 min. This step further helped in eliminating any remaining moisture and creating suitable conditions for CO_2_ adsorption. The pressure in the system was regulated using a pressure gauge, ensuring it was at the desired level. Similarly, the temperature was controlled using a thermostat, maintaining it at the appropriate value required for the experiment. Once the temperature of the reactor dropped to 25 °C, the CO_2_ gas was introduced into the reactor. This marked the beginning of the adsorption process where the CO_2_ gas interacts with the prepared adsorbent material. By following this step-by-step procedure, the adsorbent was effectively prepared, ensuring optimal conditions for dehumidification and pre-adsorption of CO_2_. The CO_2_ gas was obtained from a high-purity cylinder and passed through a regulator to achieve the desired conditions. It was then directed into a mixing vessel, where the pressure of the gas was adjusted to match the desired pressure for the reactor.

To ensure accurate data collection, a computer was used to record all the experimental data at one-second intervals. This allowed for precise monitoring of the process and analysis of the obtained results. Once the gas in the mixing tank reached the desired pressure, it was transferred to the reactor. The gas valve was then closed to prevent any further flow of gas. This ensured that the reactor operated under controlled conditions and allowed for accurate evaluation of the adsorption process.

To determine the extent of CO_2_ adsorption, a gas analysis method was employed. The adsorption rate of CO_2_ was measured through a material balance approach in the gas phase. This involved quantifying the amount of CO_2_ that was adsorbed by the material by comparing the initial and final concentrations of CO_2_ in the gas phase. By employing both the gas analysis method and the material balance approach, the adsorption of CO_2_ could be accurately assessed. The gas analysis provided insights into the change in CO_2_ concentration, while the material balance approach enabled the determination of the rate at which CO_2_ was being adsorbed by the material. These analytical techniques allowed for a comprehensive understanding of the CO_2_ adsorption process, facilitating the evaluation of the effectiveness of the adsorbent material and the optimization of the adsorption conditions. To determine the adsorption capacity, the equation for Eq. (1) incorporates the variables of pressure (P) and temperature (T).

1 $$q=({m}_{i}-{m}_{f})/w=(V{M}_{w}/Rw)\left({P}_{i}/{Z}_{i}{T}_{i}-{P}_{f}/{Z}_{f}{T}_{f}\right)$$

The symbols used in the equation are as follows: T represents the temperature, V symbolizes the volume of the reactor, P represents the pressure of the gas, Z denotes the compressibility factor, w signifies the weight of the sorbent, R represents the universal gas constant, Mw symbolizes the molecular weight of CO_2_, f represents the final state, and i denotes the initial state. The compressibility factor, Z, is calculated using the Virial equation (Eq. 2), regardless of coefficients beyond the second coefficient. The Tsonopolos equation (Eqs. 3–5) is utilized to calculate the Virial second coefficient^32^.

2 $$Z=1+\frac{BP}{RT}$$

3 $$\frac{B{P}_{c}}{R{T}_{c}}={F}^{(0)}\left({T}_{R}\right)+\omega {F}^{(1)}\left({T}_{R}\right)$$

4 $${F}^{(0)}\left({T}_{R}\right)=0.1445-\frac{0.330}{{T}_{R}}-\frac{0.1385}{{T}_{R}^{2}}-\frac{0.0121}{{T}_{R}^{3}}-\frac{0.000607}{{T}_{R}^{8}}$$

5 $${F}^{(1)}\left({T}_{R}\right)=0.0637+\frac{0.331}{{T}_{R}^{2}}-\frac{0.423}{{T}_{R}^{3}}-\frac{0.008}{{T}_{R}^{8}}$$

### Response surface methodology

To improve the process conditions for carbon dioxide adsorption using M-MWCNT, an experimental design was conducted. The response surface methodology (RSM) was employed to identify the optimal operating conditions based on the experimental findings. RSM is a powerful technique that combines statistical and mathematical methods, initially introduced by Box and Wilson, to model, analyze, modify, and optimize diverse processes^58,59^. The objective of this research was to achieve optimal conditions for maximizing CO_2_ adsorption by manipulating three independent variables. Table 1 provides detailed information regarding the specific ranges assigned to each independent variable (Pressure, time of adsorption process, and temperature), enabling a comprehensive exploration of the parameter space.

**Table 1:** The specific ranges assigned to each independent variable.

Factor	Name	Units	Minimum	Maximum	Coded low	Coded high	Mean	SD
A	Pressure	bar	4.0	10.0	− 1 ↔ 4.0	+ 1 ↔ 10.0	6.99	2.24
B	Time	Sec	0.000	5292.0	− 1 ↔ 0.0	+ 1 ↔ 5292.0	2608.25	1581.89
C	Temperature	K	298.0	328.0	− 1 ↔ 298.0	+ 1 ↔ 328.0	312.64	11.23

When determining realistic levels for each independent factor (pressure, temperature, and time) in a study, several considerations need to be taken into account. These considerations are influenced by the nature of the study, its objectives, and any limitations or constraints involved. Some of the factors to consider include:

- *Feasibility* It is important to consider the practicality and feasibility of the selected levels for each factor. Can they be achieved and maintained within the experimental setup? Are the resources available to reach those levels? It is important to select levels that are achievable and realistic within the given constraints.
- *Range* The selected levels should cover a broad range to ensure that the study captures the possible effects of the factors. This range should include both low and high levels to account for any potential nonlinear relationships or thresholds.
- *Relevance* The levels selected should be relevant to the study's objectives. They should be within the range of the actual values encountered in the real-world application or process being studied. This ensures that the findings of the study can be directly applied to real-life situations.
- *Previous research and literature* Reviewing existing literature and previous research in the field can provide valuable insights into the appropriate levels for each factor (Table 10). It helps to avoid redundancy and ensures that the study builds upon existing knowledge.
- *Statistical considerations* Statistical techniques, such as the design of experiments, can help determine the number of levels and their distribution. These techniques ensure that the selected levels provide sufficient power and precision to detect significant effects and relationships.

Aligning the selection of levels with the study's objectives ensures that realistic conditions are created to investigate the effects of the independent factors accurately. This alignment also enables researchers to draw valid conclusions and make practical recommendations based on their findings. By considering these factors, we can ensure that the experiments we design for our specific study are realistic, applicable, and reliable.

In the context of response surface methodology (RSM), the domain of independent factors refers to the range or set of values that can be assigned to each independent factor in a study. The domain represents the feasible and meaningful range within which the factors can be varied to conduct the experiments. The criteria that guide the inclusion or exclusion of independent factors in RSM depend on several factors, such as the research objectives, prior knowledge, available resources, and experimental design considerations. Some common criteria include:

- *Relevance* Factors should be relevant to the research problem being investigated. They should have a potential influence on the response variable of interest or be related to the phenomenon under study.
- *Controllability* Factors that are controllable or can be manipulated within the experimental setup are preferred. Controllable factors allow researchers to actively change their values and observe the corresponding effects on the response variable.
- *Fixed or random effects* Factors can be classified as fixed or random effects based on the research design. Fixed factors are those for which specific levels are chosen by the experimenter. Random factors are those whose levels are considered as a random sample from a larger population. The inclusion or exclusion of these factors depends on the experimental design and the objectives of the study.
- *Resource constraints* The availability of resources, such as time, cost, and availability of materials or equipment, can influence the selection of factors. Practical limitations may require researchers to select a subset of factors that can be reasonably handled within the available resources.
- *Statistical significance* Factors that are expected to have a significant impact on the response variable based on prior knowledge or literature can be included in the RSM model. Statistical techniques, such as analysis of variance (ANOVA), can help identify significant factors and remove non-significant ones from the model.
- *Interactions* Factors that are suspected to interact with each other, meaning their combined effect is different from their individual effects, are considered for inclusion. Interactions can provide valuable insights into the system's behaviour and understanding.

It is important to note that the criteria for including or excluding factors should be determined before conducting the experiments and should be based on a solid understanding of the research problem and the underlying factors affecting the response variable.

The prioritization or choice of independent factors, such as pressure, temperature, and time, in determining their potential impact on the dependent variable, CO_2_ capture, is typically based on scientific principles and prior research. Here are some factors that contribute to their selection:

- *Thermodynamics and chemical principles* The selection of pressure and temperature as independent factors is often guided by the fundamental principles of thermodynamics and chemistry. These factors are known to influence the behavior of gases, including CO_2_, and their interactions with various materials used for capture.
- *Literature review and theoretical models*^3,47^ Researchers usually conduct a thorough literature review to understand previous studies on CO_2_ capture and the factors affecting it. This helps in identifying the key independent variables that have been reported as significant in previous research. Modelling and theoretical calculations may also aid in suggesting influential factors.
- *Experimental observations and correlations* Experimental studies investigating CO_2_ capture under varying conditions provide insights into the impact of pressure, temperature, and time. These experiments often involve studying different capture technologies, such as adsorption, absorption, or membrane separation, and measuring CO_2_ capture efficiency under various conditions. Statistical analysis of experimental data can help identify the independent factors that exhibit a significant influence on CO_2_ capture.
- *Computational simulations* Computational modeling and simulations are valuable tools to understand the impact of independent factors on CO_2_ capture.

In this context, a quadratic model and regression equations were derived based on experimental data. In response surface methodology (RSM), the quadratic model serves as a mathematical representation of the relationship between a response variable and independent variables. Its purpose is to analyze and optimize complex systems by examining the impact of independent variables on the response variable. The quadratic model encompasses linear, quadratic, and interaction terms. The linear terms indicate the direct effect of each independent variable, while the quadratic terms account for any curvature in the relationship. The interaction terms capture the combined effects of the independent variables. The quadratic model in RSM can be mathematically expressed as:

6 $$ y = \beta_{0} + \mathop \sum \limits_{i = 1}^{k} \beta_{i} X_{i} + \mathop \sum \limits_{i = 1}^{k} \mathop \sum \limits_{j = 1}^{k} \beta_{ij} X_{i} X_{j} \mathop \sum \limits_{i = 1}^{k} \beta_{ii} X_{i}^{2} + \varepsilon $$

7 $$ Y \, = \, \beta_{0} + \, \beta_{1} X_{1} + \, \beta_{2} X_{2} + \, \cdots \, + \, \beta_{ii} X_{1}^{2} + \, \beta_{jj} X_{2}^{2} + \cdots \, + \, \beta_{ij} X_{1} X_{2} + \, \varepsilon $$

Here, Y represents the response variable, β_0_ is the intercept, β_1_, β_2_, and so on are the coefficients for the linear terms, β_ii_ and β_jj_ represent the coefficients for the quadratic terms, β_ij_ denotes the coefficients for the interaction terms, X_1_, X_2_, and so forth are the independent variables, and ε signifies the error term^31,60^. Determining the coefficients of the quadratic model involves collecting experimental values and performing regression analysis. This analysis estimates the coefficients that best fit the collected data, facilitating prediction and optimization of the response variable. In practice, the quadratic model is utilized to generate response surface plots, which provide a visual representation of the relationship between independent variables and the response variable. These plots aid in identifying the optimal settings for independent variables that optimize or minimize the response variable.

### ANN modeling

Artificial Neural Network (ANN) modeling is a fascinating field of study that aims to mimic the intricate workings of the human brain^61,62^. ANN models consist of interconnected artificial neurons that can process and analyze complex data, enabling them to make predictions, recognize patterns, and solve intricate problems. One of the most remarkable aspects of ANN modelling is its versatility, as it can be applied to a wide range of domains. By training the neural network on labeled data, it can learn from examples and make accurate predictions on unseen data^32^. The process of normalization is used to scale the values of a variable to a specific range, typically between 0 and 1^63^. This allows for fair comparisons and analysis of different variables that may have different scales or units. One of the most commonly used normalization methods involves linearly mapping the data onto a specified range. This method converts any value of a variable x according to the following formula:

8 $$ x_{n} = \left( {\frac{{x - x_{\min } }}{{x_{\max } - x_{\min } }}} \right) \times (r_{\max } - r_{\min } ) + r_{\min } $$

The common method of normalization involves mapping the original data, denoted as "x", to a normalized value, denoted as "xn". This mapping is done using the maximum and minimum values of the respective variables, denoted as "min" and "xmax", respectively. In this formula, "x" represents the original data, "xmin" represents the minimum value of the variable, and "xmax" represents the maximum value of the variable^64^.

The main objective of constructing an Artificial Neural Network (ANN) is to uncover the relationship between input and output data^60^. ANNs simulate the behaviour of the human brain using interconnected layers of neurons. The hidden layers, positioned between the input and output layers, process information and extract meaningful patterns. Determining the optimal number of neurons and hidden layers involves iterative experimentation and fine-tuning. The goal is to strike a balance between capturing complexity and avoiding overfitting. By finding the right configuration, ANNs can accurately predict and provide insights into the underlying patterns of the system being modelled^65^. The accuracy and performance of the model based on artificial neural networks were evaluated using several statistical criteria. These criteria included the mean squared error (MSE), correlation coefficient (R^2^), and accuracy between the predicted values and actual values^33^. To calculate the MSE and R^2^, the following equations were used^32^:

9 $$ MSE = \frac{1}{N}\sum\limits_{i = 1}^{N} {(Y_{predicted} } - Y_{real} )^{2} $$

10 $$ R^{2} = \sum\limits_{i = 1}^{N} {\frac{{(Y_{predicted} - Y_{real} )^{2} }}{{(Y_{predicted} - Y_{mean} )^{2} }}} $$

These calculations provide insights into the accuracy and performance of the model and help evaluate its effectiveness. In an MLP (Multi-Layer Perceptron), the activation function used for the input layer is often chosen based on the specific problem and input data. One commonly used activation function for the input layer is the Tanh-sigmoid function. As for the output layer, it typically employs a linear activation function. This linear function allows the MLP to directly output the computed values without any additional non-linear transformation. Mathematical and graph symbols of activation functions are indicated in Table 2.

**Table 2:** Mathematical and graph symbols of activation functions^66^.

Transfer function	Hidden layer	Out layer
Graph of activation function		
Equations	$a = \tan sig(n) = \frac{2}{{(1 + \exp ( - 2*n)^{ - 1} )}}$	$a = purelin(n) = n$

Here are some of the technical reasons for using ANN modelling in this study:

- *Complex and non-linear relationships* ANN modelling allows for the exploration of complex and non-linear relationships between various parameters in the CO_2_ capture process. The behaviour of M-MWCNTs in terms of CO_2_ adsorption and desorption is influenced by multiple factors, such as temperature and pressure. ANN can capture these non-linear relationships and provide accurate predictions.
- *Data-driven approach* ANN modelling is a data-driven approach, which means that it leverages the available experimental data to train the model. In this study, ANN can utilize the experimental data on CO_2_ adsorption to establish the relationships between these variables and provide a predictive model.
- *Prediction capability* ANN modelling can learn from the available data and generate predictions for CO_2_ capture performance based on the learned patterns. It can be used to predict the optimal conditions for CO_2_ adsorption, as well as the efficiency and capacity of M-MWCNTs for CO_2_ capture.
- *Optimization and parameter tuning* ANN modelling can also be used for optimization and parameter tuning purposes. By utilizing various optimization algorithms, ANN can find the optimal combination of parameters and conditions to maximize the CO_2_ capture efficiency of M-MWCNTs. This can help in designing and improving the performance of CO_2_ capture systems.

Overall, ANN modelling is employed in this study to better understand the behaviour of M-MWCNTs in CO_2_ capture processes and provide accurate predictions. It allows for the exploration of complex relationships and utilizes the available experimental data to generate useful insights and predictions^67^.

### Multilayer perceptron

Artificial neural networks, like MLP, offer various forms for solving problems. MLP stands out for its high-quality models, simplicity of implementation, and efficient training time. MLP networks consist of three layers: the input, hidden, and output layers, each containing multiple neurons. Neurons within each layer are connected to the next layer through weighted connections^66^. With the inclusion of a hidden layer and sufficient neurons, MLP can fit any input–output problem. This flexibility and effectiveness make MLP networks a popular choice in the field of artificial neural networks. In general, neural networks can be expressed as Eq. (11).

11 $$ y = f_{2} \left( {\sum\limits_{j = 1}^{N} {w_{j} f_{1} \left( {\sum\limits_{i = 1}^{n} {h_{ij} X_{i} + b_{j} } } \right)} } \right) + b_{O} $$

In this network, we have the weight matrix represented by h_ij_, the bias vector represented by bj, and the activation function represented by f_1_ for the hidden layer. Similarly, we have the weight vector represented by w_j_, the bias scalar represented by b_o_, and the activation function represented by f_2_ for the output layer^68^.

### Characterization

The features of the produced samples were analyzed qualitatively and quantitatively using various characterization techniques. The volumetric micro-politics ASAP2020 (Micromeritics Corp., USA) adsorption analyzer was used to measure the N_2_ isotherm models at 77 K. Before performing the adsorption–desorption study, the materials were autoclaved for five hr at a temperature of around 155 °C to maintain a constant mass under cyclic vacuum pressure. The specific surface area was calculated using the multipoint (BET) method at a relative operating pressure of p/p_0_ = 0.055–0.20, which implies Brunauer–Emmett–Teller and the total pore volumes were measured at p/p_0_ = 0.955. Using the Barrett-Joyner-Halenda (BJH) approach, the mesopore surface area, porosities, and pore size distribution could all be calculated. The Dubinin-Astakhov (DA) method is used to calculate micropore size, and the t-method is used to calculate the micropore surface area and volume. The potassium bromide (KBr) disc technique was used for FTIR spectroscopy on a Perkin-Elmer Spectrometer in the 500–4000 cm^–1^ band. FESEM was seen on a Nanosem 450 microscope.

## Results and discussions

### The characterization of adsorbent

**Figure 4:**
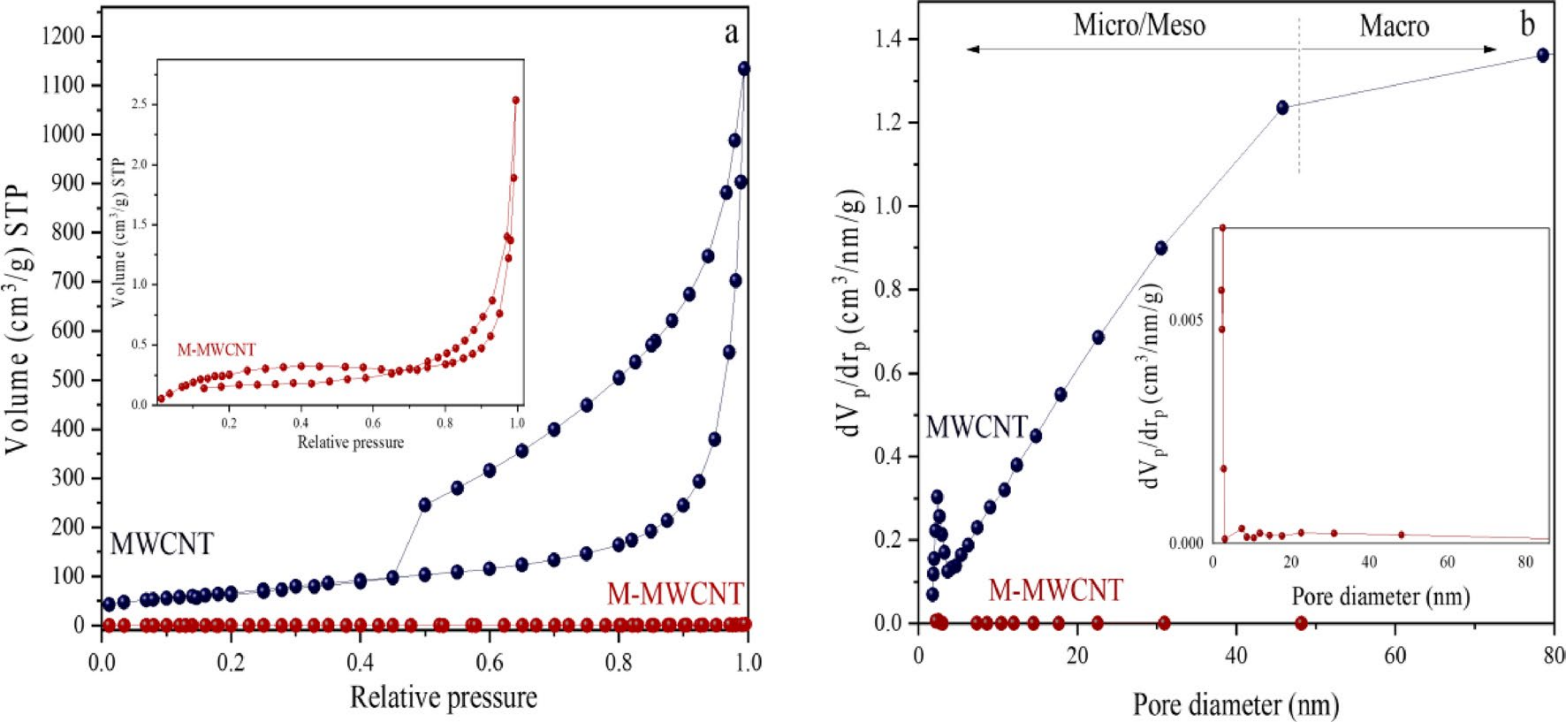
The N_2_ adsorption/desorption of MWCNT and M-MWCNT^45^.

Gas sorption on carbon nanotubes (CNTs) can occur in both the interstitial channel and the inside of the tubes. To evaluate the porous properties of the produced materials, nitrogen gas adsorption and desorption tests were conducted. The results are shown in Fig. 4a^69^. The porosity size distribution (PSD) is displayed in Fig. 4b. In both samples, the volume of N_2_ adsorption increases exponentially with pressure in the relative pressure range of 0.5 < P/P_0_ < 0.9, which corresponds to sorption on mesoporous and macroporous materials. For relative pressures > 0.9, there is a rapid increase in N_2_ sorption due to capillary condensation in the mesopores and macropores. The findings indicate that the modified multi-walled carbon nanotubes (MWCNTs) with a specific surface area of 11 m^2^/g exhibit greater sorption compared to the sample with a specific surface area of 240 m^2^/g. Analysis of the energy-dispersive X-ray spectroscopy (EDS) results presented in Table 1 reveals a 10% increase in the oxygen content in the modified MWCNTs. The excess oxygen observed can potentially be attributed to the utilization of NaOH or HNO_3_ during the surface modification procedure.

**Figure 5:**
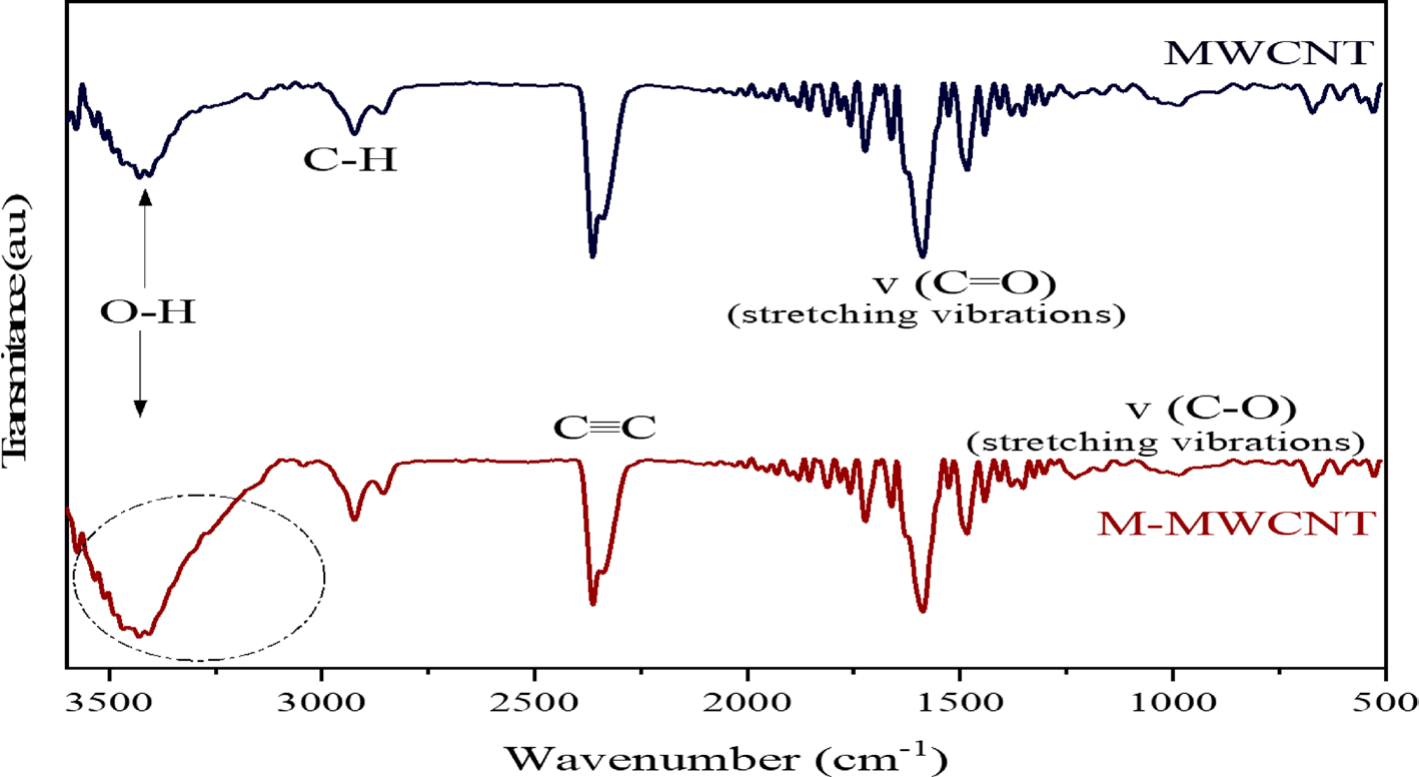
The FTIR analysis of MWCNT and M-MWCNT^45^.

FTIR analysis is a powerful technique used to identify functional groups and chemical bonding on the surface of materials. In Fig. 5, the FTIR spectra of the two synthetic carbon nanotubes (CNTs) are shown. These spectra reveal the stretching absorption bands of phenolic hydroxyl or alcoholic hydroxyl groups (-OH) that are associated with intermolecular hydrogen bonding. This absorption band is observed at a wavenumber of 3424 cm^–1^, as determined by the analyses^70^.

The intensity of the absorption peak indicates that the modified CNT sample contains a higher concentration of –OH groups compared to the pure MWCNT sample. This increase in –OH groups is a result of the alkaline pickling process using NaOH. Both synthetic CNT samples exhibit similar peaks at approximately 2900 cm^–1^ and 2850 cm^–1^. These peaks correspond to asymmetric and symmetric C-H methylene stretching vibrations, respectively. These findings provide information about the presence of specific chemical bonding and functional groups on the surface of the synthetic CNTs. The presence of a peak at approximately 2362 cm^–1^ indicates the presence of C≡C (alkynes) stretching vibrations. Peaks at 1700 cm^–1^ and 1590 cm^–1^correspond to stretching vibrations of –COOH and C=O in carbonyl and carboxylic groups, respectively^71^. This suggests the presence of these functional groups in the sample. Additionally, the absorbance band at 1030 cm^–1^ correlates with C–O flexural vibrations in carboxylate and ether group structures. Due to the abundance of oxygenated functional groups (–OH), the modified sample's surface carries a negative charge. Hence, these spectral features and oxygenated groups contribute to the surface properties of the sample.

**Figure 6:**
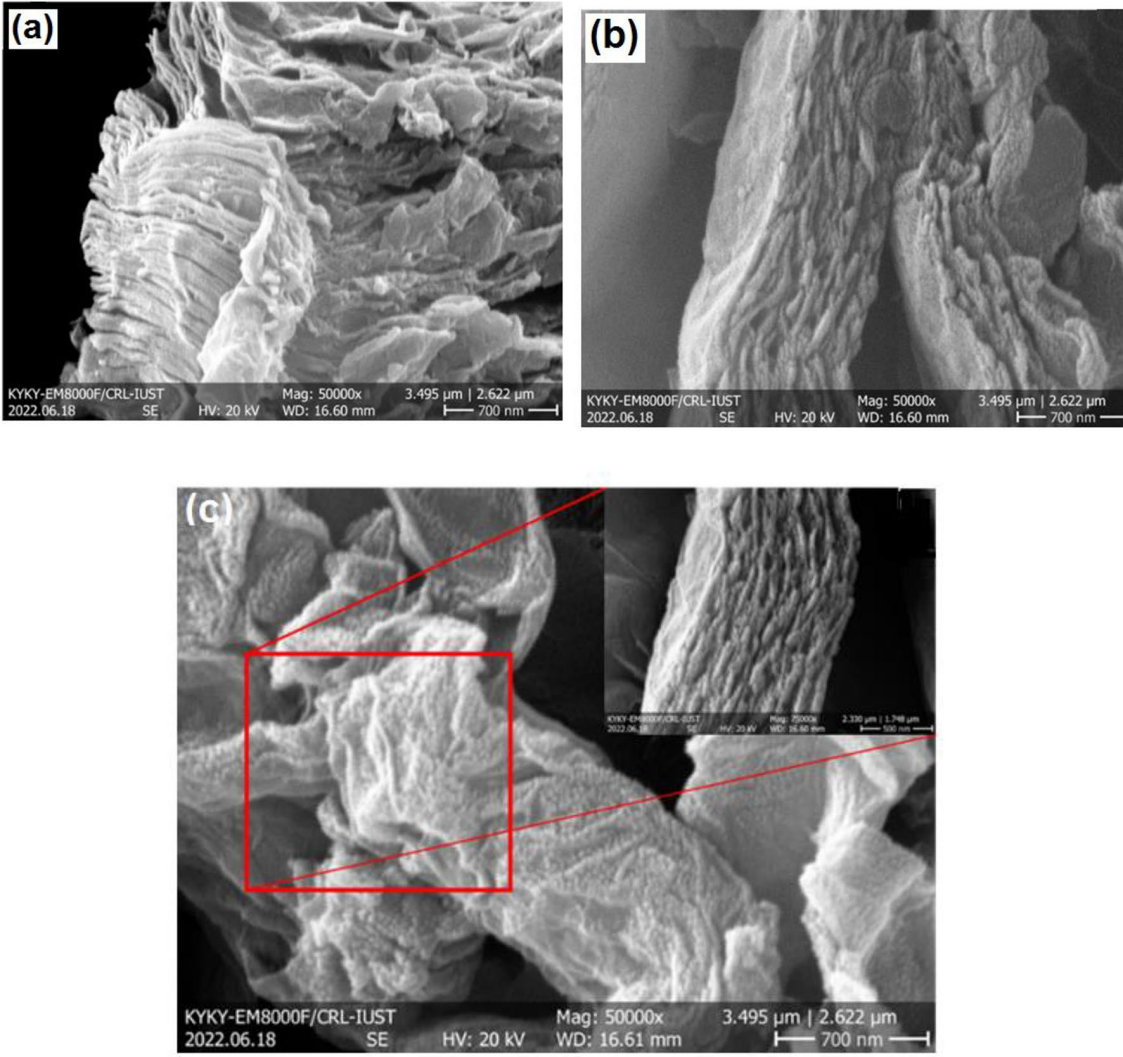
The FESEM analysis of (**a**) MWCNT and (**b, c**) M-MWCNT^45^.

FESEM was employed to analyze the surface features and morphology of multi-walled carbon nanotubes (MWCNT) before and following modification. An image of pristine MWCNT is depicted in Fig. 6a, while Fig. 6b exhibits M-MWCNT. The FESEM micrograph reveals the presence of highly compact MWCNT structures that resemble upright forests, growing perpendicular to the substrate. After subjecting the MWCNTs to strong acids (HNO_3_ and HCl), it can be observed that the walls of the tubes have not been destroyed. This is likely due to the MWCNTs' robust nature and their ability to resist damage caused by acid treatment, thanks to the van der Waals forces that hold them together^35,72,73^. Interestingly, there is a slight aggregation of O–H groups that partially cover the exterior surface of the MWCNTs (as shown in Fig. 6b). Furthermore, these O–H groups are evenly dispersed throughout the nanotubes (Fig. 6c)^45^.

The presence of aggregated O–H groups on the exterior surface of the MWCNTs suggests that functionalization occurred during the acid treatment. Functionalization refers to the attachment of chemical groups or molecules to the surface of a material, which can modify its properties or enable specific interactions. The evenly dispersed distribution of the O–H groups suggests that the functionalization process was uniform and thorough. This is important as it indicates that the acid treatment was effective in introducing the functional groups onto the MWCNTs. The robust nature of the MWCNTs, which allows them to resist damage caused by acid treatment, can be attributed to the strong van der Waals forces between the carbon atoms in the nanotubes. These forces are responsible for holding the carbon atoms together and give MWCNTs their unique mechanical properties. Overall, the observation of partially covered MWCNTs with aggregated O–H groups indicates successful functionalization and highlights the robust nature of MWCNTs. This information can be valuable for understanding the behaviour and potential applications of MWCNTs in various fields. Multi-walled carbon nanotubes (MWCNTs) possess distinctive attributes such as elongated structures and smooth surfaces. However, when exposed to strong acids, these nanotubes undergo functionalization, leading to a perceived shorter length and enhanced orderliness. It is believed that strong acid pickling targets the breakdown site of M-MWCNTs, consuming oxidized vacancies. Additionally, some carbon–carbon double bonds (C = C) are cleaved, resulting in the formation of shorter nanotubes. This process is consistent with the observed average pore diameter. In simpler terms, the strong acid treatment modifies the MWCNTs, making them appear shorter and more organized by removing certain components and breaking specific bonds^38,45^.

### Isotherm modeling

**Figure 7:**
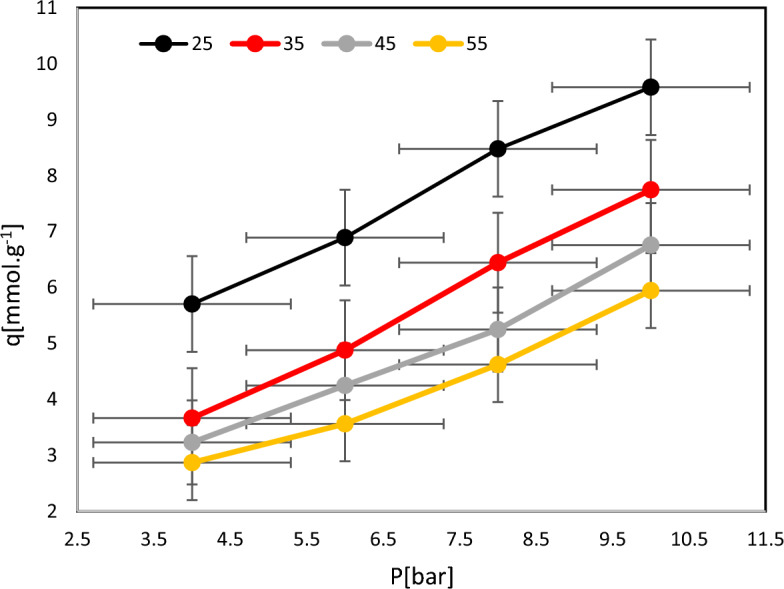
CO_2_ adsorption equilibrium isotherms of M-MWCNT adsorbent.

For scaling up and designing the adsorption process, isotherm studies offer crucial information. Adsorption equilibrium studies, particularly at the equilibrium stage, give detailed information about the adsorption of gas between the adsorbent surface and the adsorbate^74^. The diagram of uptake capacity against pressure is shown in Fig. 7. At a temperature of 25 °C and pressure of 10 bar, the study found that the maximum CO_2_ uptake value is 9.57 mmol/g. However, as the operating temperature increases, the CO_2_ uptake decreases. This decrease can be attributed to the effect of increased temperature on the behavior of CO_2_ molecules and the stability of the system. When heat is added to the system, it increases the kinetic energy of the CO_2_ molecules, causing them to move more rapidly. This increased molecular motion makes it more difficult for CO_2_ molecules to adhere to the surface of the substance and be adsorbed. As a result, the overall CO_2_ uptake decreases as the temperature rises. Additionally, the elevated temperature can also lead to the formation of unstable molecules within the system. These unstable molecules are less likely to interact with the substance's surface and undergo adsorption. This further contributes to the reduction in CO_2_ uptake at higher temperatures. It is important to consider these temperature effects when designing processes for CO_2_ capture and storage. Understanding the temperature-dependent behavior of CO_2_ adsorption can help optimize conditions to achieve higher uptake values and improve the efficiency of CO_2_ capture technologies^75,76^. Adsorption of CO_2_ is a physical process known as physisorption, where molecular diffusion and surface adsorption energy are affected by temperature^32,77^. As temperature increases, molecular movement and diffusion become more prominent, leading to an increase in gas instability and desorption. On the other hand, increasing pressure enhances the amount of CO_2_ adsorption. This is because higher pressures result in a higher concentration of CO_2_ molecules in the surrounding environment, increasing the chances of them coming into contact with the substance's surface and being adsorbed. However, it is important to note that the adsorption process is exothermic, meaning it releases heat. As temperature rises, the increased thermal energy can weaken the attractive forces between the CO_2_ molecules and the adsorbent surface. This, in turn, reduces the adsorption capacity and leads to a decrease in the amount of CO_2_ adsorbed as temperature increases desorption^78^.

**Figure 8:**
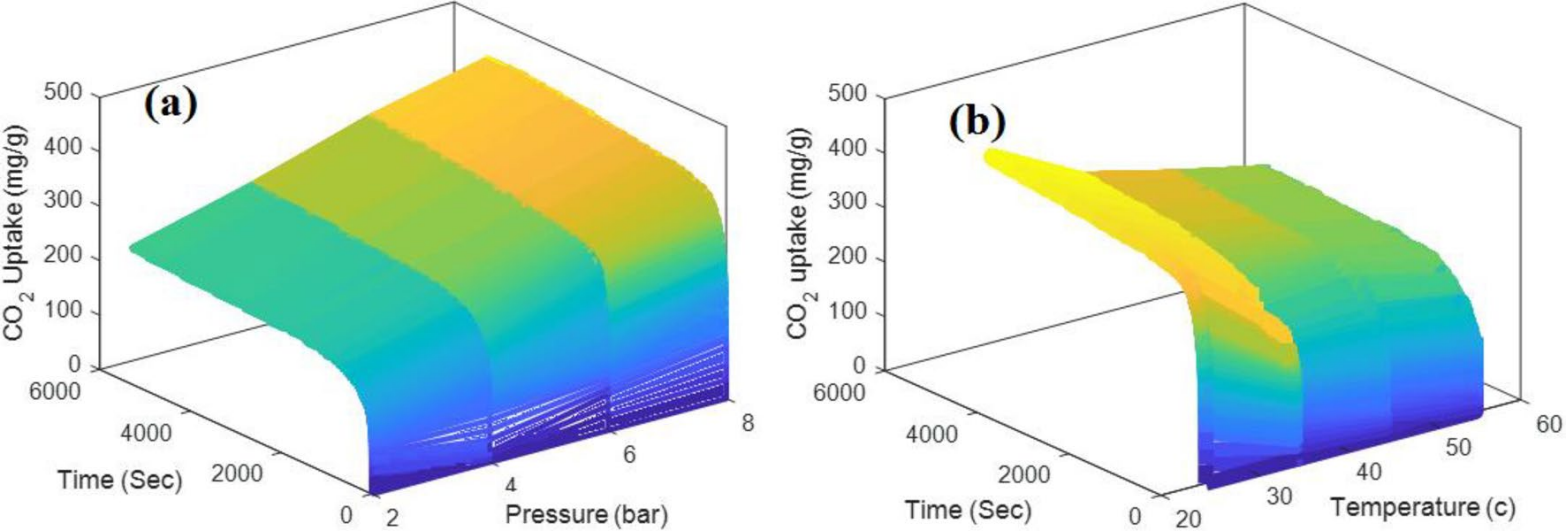
(**a**) The effect of time, pressure on CO_2_ uptake, (**b**) the effect of and temperature and time on CO_2_ uptake.

**Figure 9:**
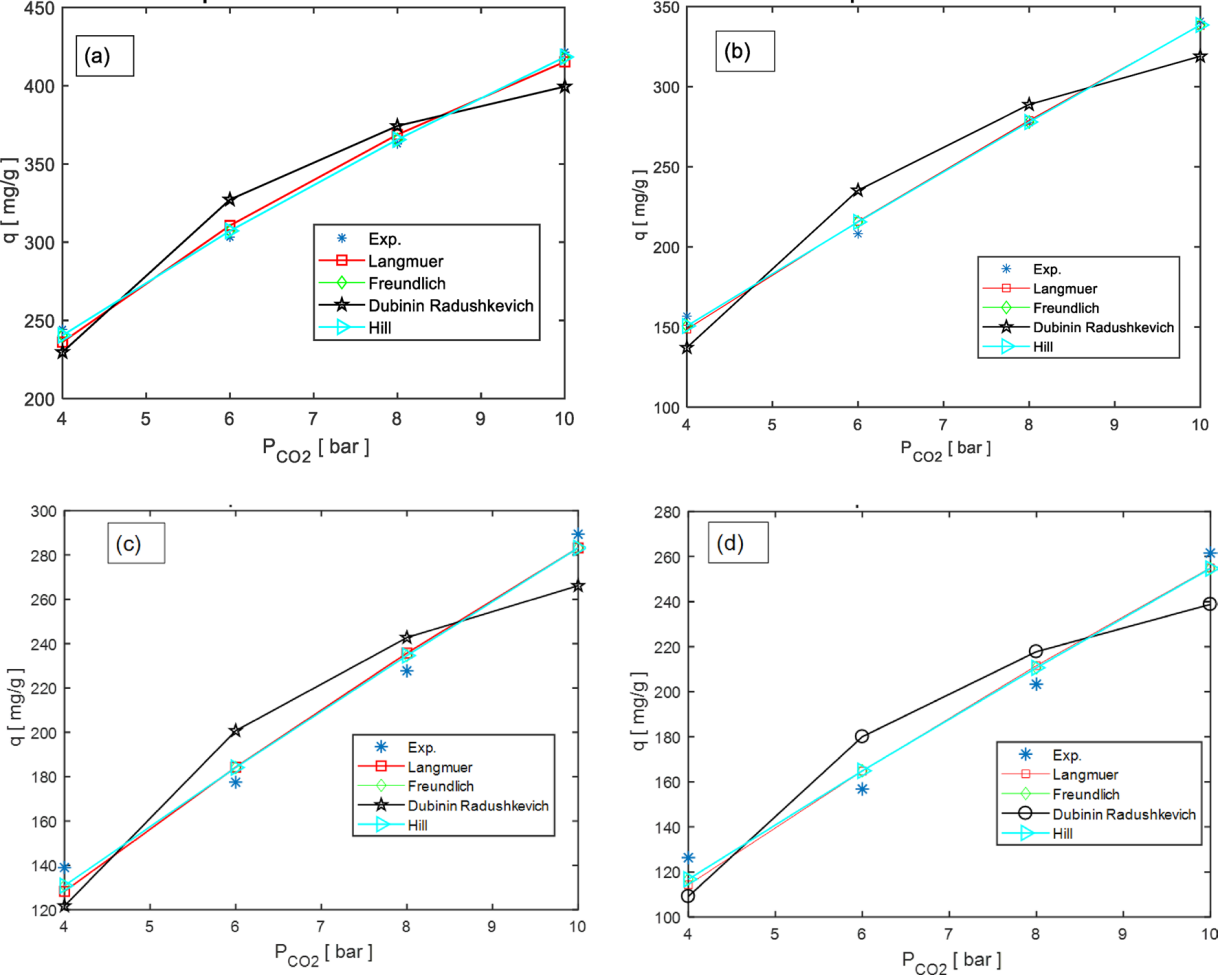
Carbon dioxide adsorption experimental on the M_MWCNT at (**a**) 25, (**b**) 35, (**c**) 45, and (**d**) 55 °C.

The effect of three parameters on gas capture is indicated in Fig. 8. This implies that as the pressure is raised, more of the substance is being adsorbed onto the material being studied. The carbon dioxide adsorption experimental on the M-MWCNT at 25, 35, 45, and 55 °C indicated in Fig. 9. The isotherm model parameters for the gas adsorption on the modified M-MWCNT at 25, 35, 45, and 55 °C are shown in Table 3. In the current study, the four isotherm models (Langmuir, Freundlich, Hill, and Dubinin–Radushkevich (D-R)) have been used to fit the empirical results. Figure 9 displays the plotted models at a consistent temperature and pressure range of 4 to 10 bar. Furthermore, Table 4 provides the model parameters and correlation coefficients (R^2^) for temperatures of 25, 35, 45 and 55 °C.

**Table 3:** Models and formulae for isotherms^32^.

Model		Parameters
Langmuir ${q}_{e}=\frac{{q}_{m}{K}_{L}{P}_{CO2}}{1+{K}_{L}{P}_{CO2}}$	${q}_{m}$	*q*_m_ is the max adsorption value of CO_2_ (mg g^−1^)
	${K}_{1}$	Langmuir equilibrium constant (bar^−1^)
	${q}_{e}$	*q*_e_ is the value of CO_2_ adsorption capacity (mg g^−1^)
	*P*	*P* is the equilibrium pressure (bar)
Freundlich ${q}_{e}={K}_{f}\times {{P}_{CO2}}^\frac{1}{n}$	${K}_{F}$	*K*_F_ is the Freundlich model constants [(mg g^−1^). (bar^−1^)^1/*n*^)]
	$n$	*n* is Freundlich isotherm constant (dimensionless)
	*q*_*e*_	*q*_e_ is the value of CO_2_ adsorption capacity (mg g^−1^)
D-R ${\text{ln}}{q}_{e}={\text{ln}}{q}_{m}-\beta .{\varepsilon }^{2}$ $\varepsilon =RTln\left[1+\frac{1}{{P}_{CO2}}\right]$	${q}_{m}$	The single-layer adsorption capacity
	$\beta$	The constant associated with adsorption energy (mol^2^ KJ^−2^)
	${q}_{e}$	The amount of CO_2_ adsorbed per unit mass of AC at equilibrium (mg g^−1^)
	$\varepsilon$	Adsorption potential (kJ mol^−1^)
	R	gas constant (8.314 J mol^−1^ K^−1^)
Hill ${q}_{e}=\frac{{q}_{{s}_{H}}{{P}_{CO2}}^{{n}_{H}}}{{K}_{D}+{{P}_{CO2}}^{{n}_{H}}}$	${q}_{s}$	***q***_*sH*_ is saturation for maximum uptake (mg/L)
	$n$	*n*_*H*_ coefficient of binding interaction
	$K_{D}$	Hill constants
	*P*	*P* is the equilibrium pressure (bar)

**Table 4:** The calculated parameters of isotherm models of CO_2_ adsorption on M-MWCNT.

Model	Parameters	T = 25 °C	T = 35 °C	T = 45 °C	T = 55 °C
Langmuir	$q_{m}$	839.466	2253.302	1452.539	1426.989
	$K_{1}$	0.0978895	0.0176594	0.0242199	0.0217598
	R^2^	0.9947	0.9967	0.9902	0.9855
	SSE	0.9047	2.1948	4.6582	5.9536
	ARE	2.1931	2.5304	4.3019	5.2964
Freundlich	$K_{F}$	104.016	44.292	40.665	35.879
	$n$	1.655	1.132	1.186	1.175
	R^2^	0.9987	0.9975	0.9923	0.9878
	SSE	0.0534	0.3769	0.8399	1.1425
	ARE	1.0815	2.0607	3.7003	4.7319
D-R	$q_{m}$	451.856	385.010	316.949	284.377
	$\beta_{D}$	2.213	3.161	2.750	2.587
	$\varepsilon_{D}$	0.475	0.398	0.426	0.440
	R^2^	0.9594	0.9553	0.9356	0.9231
	SSE	1.2205	8.8294	5.6529	5.5604
	ARE	5.5462	8.9957	10.0414	11.0573
Hill	$q_{s}$	2,869,597,890.660	6,588,025,440.243	10,732,289,633.535	12,435,000,785.664
	$n$	0.604	0.883	0.843	
	$K_{D}$	27,588,017.629	148,742,248.174	263,918,880.111	346,578,249.54
	R^2^	0.9987	0.9975	0.9923	0.9878
	SSE	0.0534	0.3770	0.8400	1.1426
	ARE	1.0815	2.0607	3.7003	4.7319

*SSE* sum squared error, *ARE* absolute relative error.

Based on the data presented in Table 4, it can be observed that the constant K_*f*_ of the Freundlich model, which represents the affinity between the adsorbate and adsorbent, decreases as the temperature increases. This could be due to factors such as weaker interactions between the adsorbent and the substance being adsorbed, or changes in the surface properties of the adsorbent material as temperature increases. This indicates that physisorption, a weaker form of interaction, is more prominent in the adsorption of CO_2_ by the adsorbent compared to chemisorption. Moreover, the decrease in adsorption capacity with higher temperatures suggests an exothermic nature of the adsorption process. The Freundlich constant n, falling within the range of 1–2, signifies the favorability of CO_2_ adsorption. When the Freundlich n constant falls within the range of 1–2, it suggests that the adsorption process is favourable and relatively strong. A value of n closer to 1 indicates a more homogeneous adsorption process, while a value closer to 2 indicates a more heterogeneous adsorption process^79^. Overall, a Freundlich n constant within this range signifies efficient and effective CO_2_ adsorption adsorbent material^80,81^. In addition, the Dubinin-Radushkevich model was used to calculate a value below 8 kJ/mol, indicating that the adsorption of gases onto the adsorbent surface is primarily a physical process. Considering the average R^2^ values of the isotherm models, the Freundlich model exhibits the highest accuracy among the others. This suggests that the adsorbent surface is heterogeneous and that the adsorption process occurs in multiple layers on the surface^32,74^.

### Kinetic modeling

Kinetic modeling in CO_2_ adsorption refers to the mathematical representation of the rate at which CO_2_ molecules are adsorbed onto a solid material, such as an adsorbent or a porous material. This modelling helps in understanding and predicting the behaviour of CO_2_ adsorption processes, which is crucial for various applications including carbon capture and storage (CCS), greenhouse gas mitigation, and gas separation. The kinetics of CO_2_ adsorption can be influenced by several factors, including the properties of the adsorbent material, temperature, pressure, and the concentration of CO_2_ in the gas phase^82–86^. The goal of kinetic modelling is to develop mathematical equations that describe the adsorption process accurately. Kinetic modelling was employed to specify residence duration and adsorption rate to augment the adsorption process. It explains the functionality and dynamics of adsorption, which are useful in practical industrial applications such as fabricated bed columns. So, in this work, several kinetic models were used. There are different approaches to kinetic modelling CO_2_ adsorption, and some commonly used models include (Table 5)^83,87^.

**Table 5:** Kinetic models.

$q_{t} = q_{e} \left( {1 - e^{{ - k_{f} t}} } \right)$	Pseudo-first-order	(12)
$q_{t} = \left( {q_{e}^{2} k_{s} t} \right)/\left[ {1 + \, q_{e} k_{s} t} \right]$	Pseudo-second-order	(13)
$q_{t} = \left( {1/\beta \, ln\left( {\alpha \beta } \right)} \right) + \left( {1/\beta {.}ln \, t} \right)$	Elovich	(14)
$q_{t} = q_{e} - (q_{e}^{(1 - n)} + ((n - 1)/m)k_{n} t^{m} )^{(1/(1 - n))}$	Fractional-order	(15)

Pseudo-first-order model: This model assumes that the rate of adsorption is directly proportional to the difference between the equilibrium adsorption capacity and the amount of CO_2_ adsorbed at any given time. It is a simple and widely used model but may not accurately represent the complex nature of CO_2_ adsorption^88^. Pseudo-second-order model: This model assumes that the rate of adsorption is proportional to the square of the difference between the equilibrium adsorption capacity and the amount of CO_2_ adsorbed. It provides a better fit to experimental data compared to the pseudo-first-order model^89^.

Elovich equation is a kinetic model that describes the behaviour of chemical reactions or processes where the rate of reaction decreases over time. It is commonly used to describe heterogeneous catalysis, adsorption, and other surface reactions. The Elovich equation suggests that the rate of reaction is not constant but decreases logarithmically with time. The term α ln(t) represents the initial fast adsorption process, while the term β represents the subsequent slower desorption process^90,91^.

Fractional order kinetics, on the other hand, refers to a type of reaction kinetics where the reaction order is a non-integer value. In traditional reaction kinetics, the reaction order is typically an integer or a fraction with a numerator of 1 (e.g., zero-order, first-order, second-order). However, in fractional order kinetics, the reaction order can be any real number, including non-integer values. Fractional order kinetics can provide a more accurate description of complex reaction systems where the reaction rate does not follow simple integer order kinetics. It is often used to model reactions involving complex mechanisms, heterogeneous catalysis, and biological systems. The fractional reaction orders can be determined experimentally by analyzing the concentration changes of reactants over time and fitting the data to the fractional order kinetics equation^92^. The fractional-order kinetic model offers a comprehensive representation of both physical and chemical adsorption processes. However, accurately predicting the kinetic parameters can be challenging. To address this issue, a common approach involves fitting experimental data to a range of predefined models and selecting the most suitable option that best matches the observed behaviour^93^.

**Figure 10:**
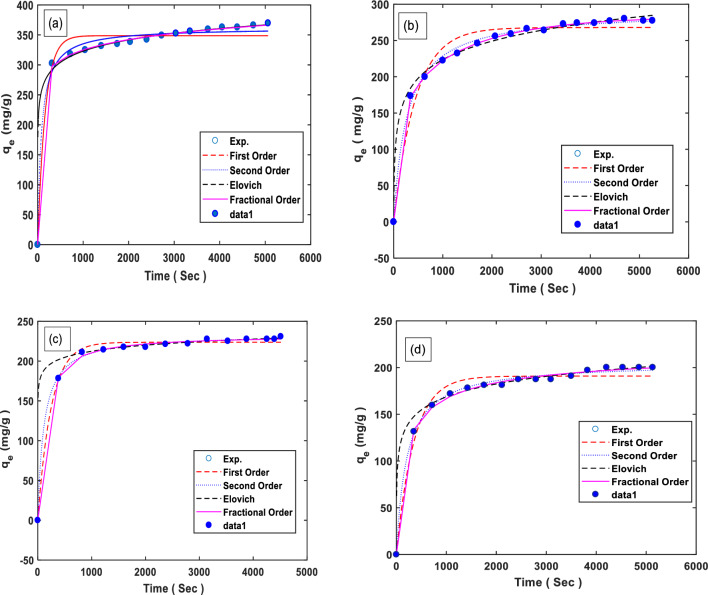
The plots of the kinetic models at (**a**) 25, (**b**) 35, (**c**) 45, and (**d**) 55 °C.

The ideal kinetic model for gas adsorption was determined by comparing the model results with the experimental data. Figure 10 displays the curves of the sorbents' kinetic models, including the Elovich, First-order, Second-order, and Fractional-order kinetic models, fitted at a temperature of 25, 35, 45, and 55 °C and a pressure of 8 bar. Upon analysis, it was observed in Fig. 10 that the Second-order and Fractional-order models exhibited a higher level of proportionality. This suggests that these models may provide a better fit to the experimental data compared to the other models^89,93,94^. The kinetic model parameters are shown in Table 6 at three different temperatures. The pseudo-first-order model describes reversible adsorption, where equilibrium is achieved at the surface of the adsorbent. On the other hand, the pseudo-second-order kinetic model incorporates the chemisorption process as a determining factor in the adsorption mechanism^93^.

**Table 6:** The calculated parameters of kinetic models of CO_2_ adsorption on M-MWCNT.

Models	25 °C	35 °C	45 °C	55 °C
First order model				
q_e_	348.703	267.837	223.701	190.878
kl	0.006	0.002	kl = 0.004	0.003
R^2^	0.98634	0.98326	0.99703	0.98728
Second order				
q_e_	362.037	291.290	233.753	205.131
K_2_	0.0000344	0.0000125	0.0000379	0.0000235
R^2^	0.99488	0.99770	0.99919	0.99784
Elovich				
Alfa	5.68877447	0.01181601	21,151.74311554	0.22880961
Beta	27.044	36.797	11.020	20.001
R^2^	0.98948	0.98426	0.97226	0.98387
Fractional order				
q_e_	1216.614	281.092	233.008	222.049
n	0.8742	0.5763	3.4170	2.0889
m	0.0856	0.3583	2.2723	0.6962
k_n_	0.0353	0.3768	0.0000	0.0001
R^2^	0.99959	0.99970	0.99929	0.99869

### Adsorption thermodynamic

**Figure 11:**
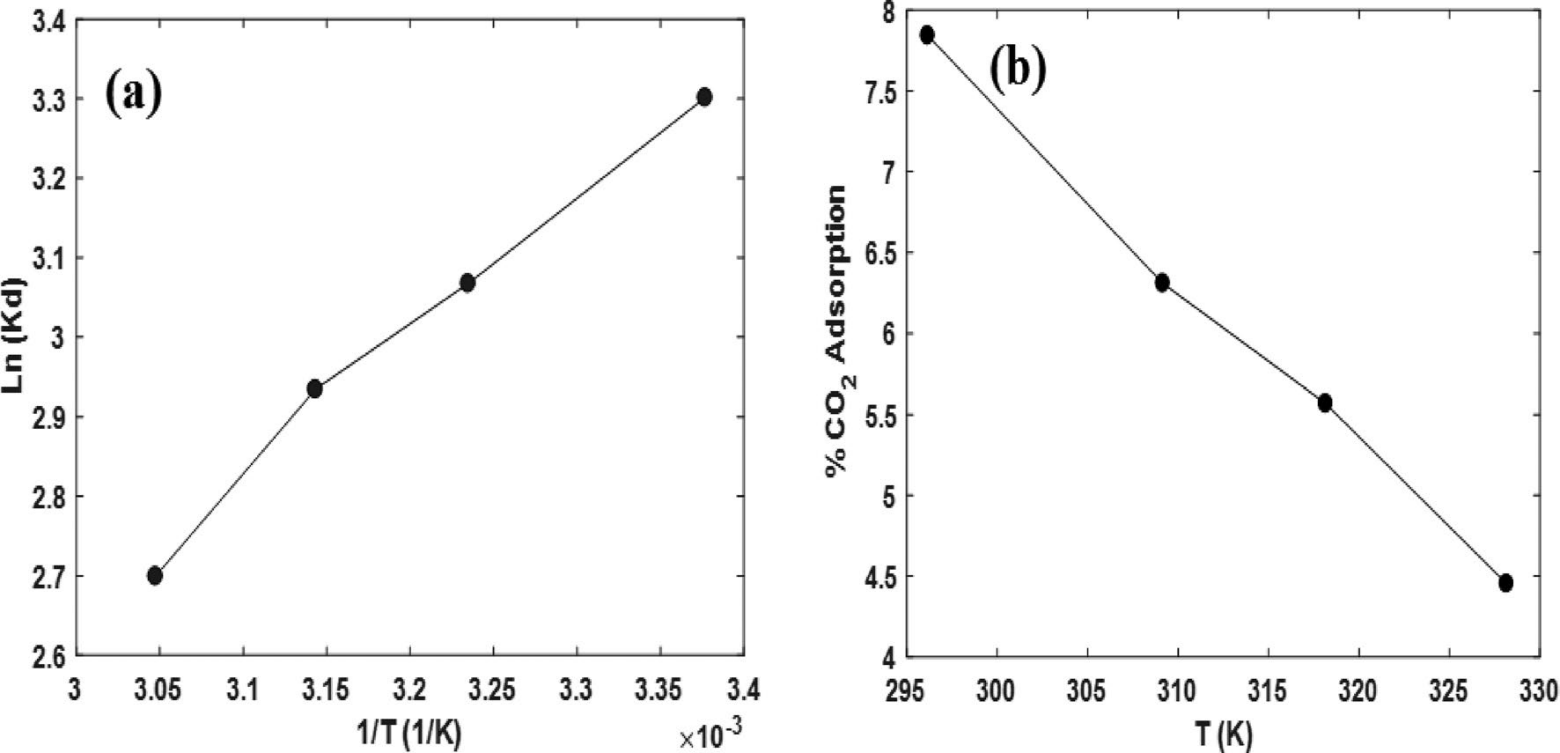
(**a**) Thermodynamic parameters of CO_2_ adsorption on M-MWCNT according to Van't Hoff, (**b**) % CO_2_ adsorption.

Figure 11a illustrates the Van't Hoff plot, which displays the equilibrium constant of Multi-walled Carbon Nanotubes (MWCNT) during the temperature range of 25–55 °C. This plot is utilized to assess the changes in entropy (*ΔS*°) and enthalpy (*ΔH*°) associated with the CO_2_ adsorption process. Table 7 provides a comprehensive overview of the thermodynamic parameters that were calculated using the experimental data collected at different temperatures, specifically within the range of 25–55 °C. These parameters are essential for understanding the thermodynamic behaviour of the system during the CO_2_ adsorption process. The calculated values of the thermodynamic parameters indicate that the process of CO_2_ adsorption is exothermic, meaning that heat is released during the adsorption process. This suggests that the adsorption of CO_2_ onto the MWCNT material is not only feasible but also spontaneous, as it occurs without the need for an external energy source. By analyzing this plot, the values for entropy change (*ΔS*°) and enthalpy change (*ΔH*°) associated with the CO_2_ adsorption process were determined. The *ΔS* value is obtained from the intercept of the plot, while the *ΔH◦*value is obtained from the slope of the plot. It is evident that as the temperature increases, the CO_2_ adsorption capacity decreases (Fig. 11b). This can be attributed to the weakening of the van der Waals bonds between the CO_2_ molecules and the adsorbent material. At higher temperatures, the increased thermal energy disrupts these bonds, leading to a reduction in the overall adsorption capacity^95,96^. This behaviour suggests that the adsorption process is exothermic, meaning that heat is released during the adsorption of CO_2_ on the adsorbent surface. Additionally, the fact that the adsorption process is primarily controlled by physical adsorption rather than chemical bonding indicates that the interaction between the CO_2_ molecules and the adsorbent surface is mainly based on weak intermolecular forces. The increase in bed temperature provides the necessary energy for the CO_2_ molecules to overcome these weak forces and detach from the adsorbent surface. This phenomenon leads to an enhancement in the adsorption efficiency as more CO_2_ molecules are released, resulting in a higher desorption capacity for the adsorbent material. Overall, these findings highlight the influence of temperature on the thermodynamics of the CO_2_ adsorption process, emphasizing the exothermic nature of the adsorption and the important role of physical adsorption mechanisms^97^.

**Table 7:** Thermodynamic parameters.

P(CO_2_)	ΔH	ΔS	ΔG (kJ/mol)			
Bar	(kJ/mol)	(kJ/mol K)	23	36	45	55
8.0	− 14.842	− 0.023	− 8.163	− 7.870	− 7.667	− 7.441

### The statistical analysis using Blank Spreadsheet Design (BSD)

The prediction of carbon dioxide adsorption was performed using Design Expert software version 13.0. To validate the experimental results, a total of 250 experiments were conducted using BSD via Stat-Ease's Design-Expert software, which is also version 13.00. The analysis utilizing Response Surface Methodology (RSM) yielded Eq. (16) for the quadratic model of CO_2_ adsorption.

16 $$ \begin{gathered} q = + 14449.46623 + 91.17477A + 0.196896B - 91.14605C + 0.000511A \times B - \hfill \\ 0.266837A \times C - 0.000478B \times C + 1.18016A^{2} - 6.73924E - 06B^{2} + 0.143866C^{2} \hfill \\ \end{gathered} $$

where A is pressure, B is time, C is the temperature.

#### ANOVA analysis

In ANOVA, the F-value is used to assess the significance of the differences between factors. It is calculated by dividing the variance between the factors by the variance within the factors. In our case, the F-value of 241.39 obtained for the model indicates its statistical significance (Table 8). The probability of obtaining such a high F-value by chance alone is exceptionally low, at only 0.01%^98^. In this analysis, model terms with a p-value less than 0.0500 are considered significant. Among the terms tested, it was found that A, B, C, AC, BC, A^2^, B^2^, and C^2^ are significant.

**Table 8:** ANOVA analysis.

Source	Sum of squares	Df	Mean square	F-value	*p* value
Model	1.529E+06	9	1.699E+05	241.39	< 0.0001
A-Pressure	7.603E+05	1	7.603E+05	1079.92	< 0.0001
B-Time	1.180E+05	1	1.180E+05	167.64	< 0.0001
C-Temperature	5.310E+05	1	5.310E+05	754.31	< 0.0001
AB	684.31	1	684.31	0.9720	0.3252
AC	10,600.47	1	10,600.47	15.06	0.0001
BC	15,398.74	1	15,398.74	21.87	< 0.0001
A^2^	5277.55	1	5277.55	7.50	0.0067
B^2^	42,622.93	1	42,622.93	60.54	< 0.0001
C^2^	48,768.59	1	48,768.59	69.27	< 0.0001
Residual	1.598E+05	227	704.00		
Cor total	1.689E+06	236			

Notably, the actual and predicted results demonstrate a remarkable resemblance, with minimal insignificant deviations observed. It was found that the predicted plot in RSM closely aligned with the actual plot, demonstrating a high R^2^ value of 0.9054. The actual values closely followed the RSM predicted values, confirming the robustness of the model with an R^2^ value of 0.9054. The remarkable R^2^ value of 0.9054 indicated a strong correlation between the RSM predicted values and the actual values, signifying the model's ability to accurately predict the outcome. The close resemblance between the RSM predicted and the actual, with an R^2^ value of 0.9054, emphasized the effectiveness of the model in capturing the underlying patterns and trends. Table 9 provides the fit statistics of the quadratic model, offering insights into its performance. The Predicted R^2^ value of 0.8899 is remarkably similar to the Adjusted R^2^ value of 0.9016, with a difference of less than 0.2. This proximity suggests that the model is highly reliable in predicting future observations, ensuring its robustness. To further evaluate the quality of the model, the Adeq Precision measure is employed. This measure compares the variation present within the data to the variation predicted by the model. In this case, an observed ratio of 64.9047 is obtained, indicating that the model's ability to navigate the design space is more than adequate. With a ratio surpassing the desirable value of 4, we can confidently assert that the model is proficient in capturing the complexity of the system and providing accurate predictions.

**Table 9:** Model validation.

SD	26.53	R^2^	0.9054
Mean	225.80	Adjusted R^2^	0.9016
C.V. %	11.75	Predicted R^2^	0.8899
		Adeq Precision	64.9047

#### Perturbation plot

**Figure 12:**
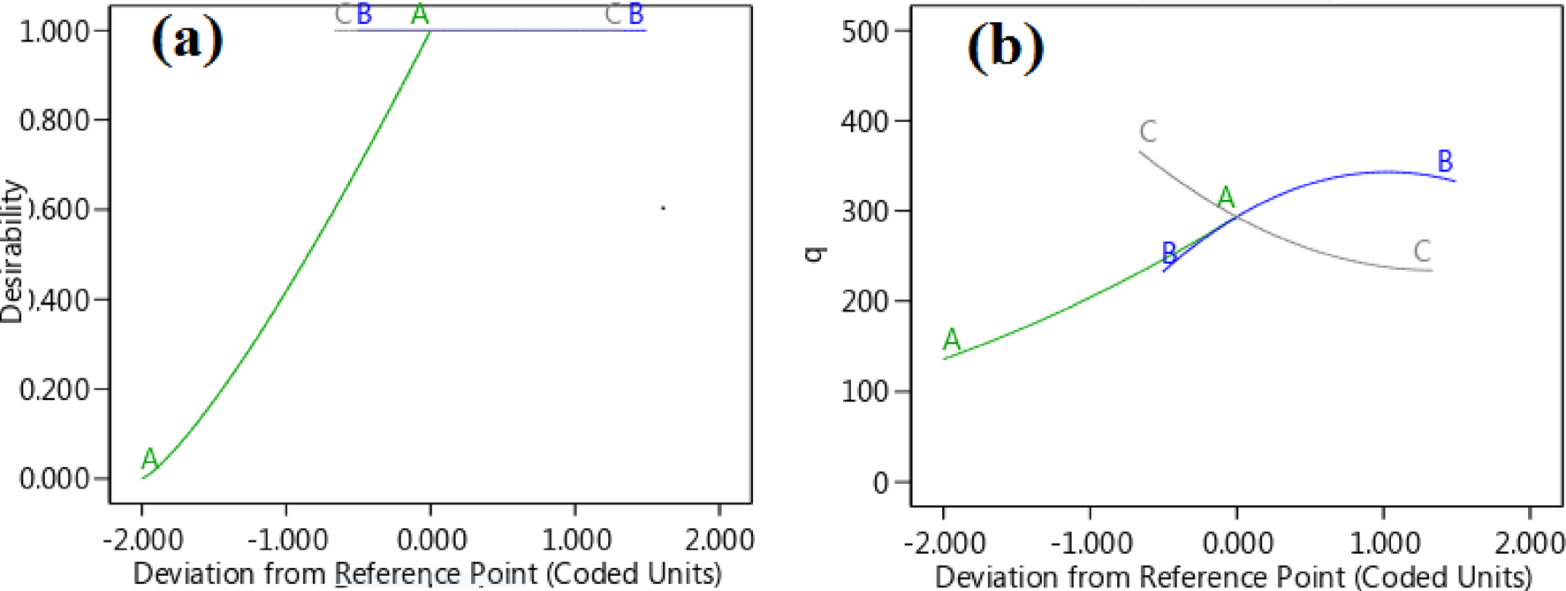
(**a**) Desirability versus deviation from a reference point; (**b**) and CO_2_ adsorption vs. deviation from a reference point (Perturbation diagram).

Figure 12 showcases a perturbation plot, providing valuable insights into the impact of three factors on the response. This graphical representation effectively visualizes the relationship between the factors under study and the system's response. By perturbing one factor while keeping the others constant, we can observe the resulting changes in the response and gain a better understanding of the system's behavior. The plot allows us to visualize the curvature of the response surface, which provides crucial information about the interactions between the factors. Each line in the plot corresponds to the perturbation of a specific factor, and its slope indicates the sensitivity of the response to that factor. Additionally, the curvature of the line helps us identify any interactions with the other factors. Upon analyzing Fig. 12a, b, we can deduce that factor A has the most significant influence on the response. This implies that any changes in factor A will have a substantial impact on the system's output. On the other hand, factor C exhibits the least influence, indicating that variations in this factor may have a relatively minor effect on the response. In the beginning, the amount of C curvature was higher, then it decreased.

#### 3D plot in RSM

**Figure 13:**
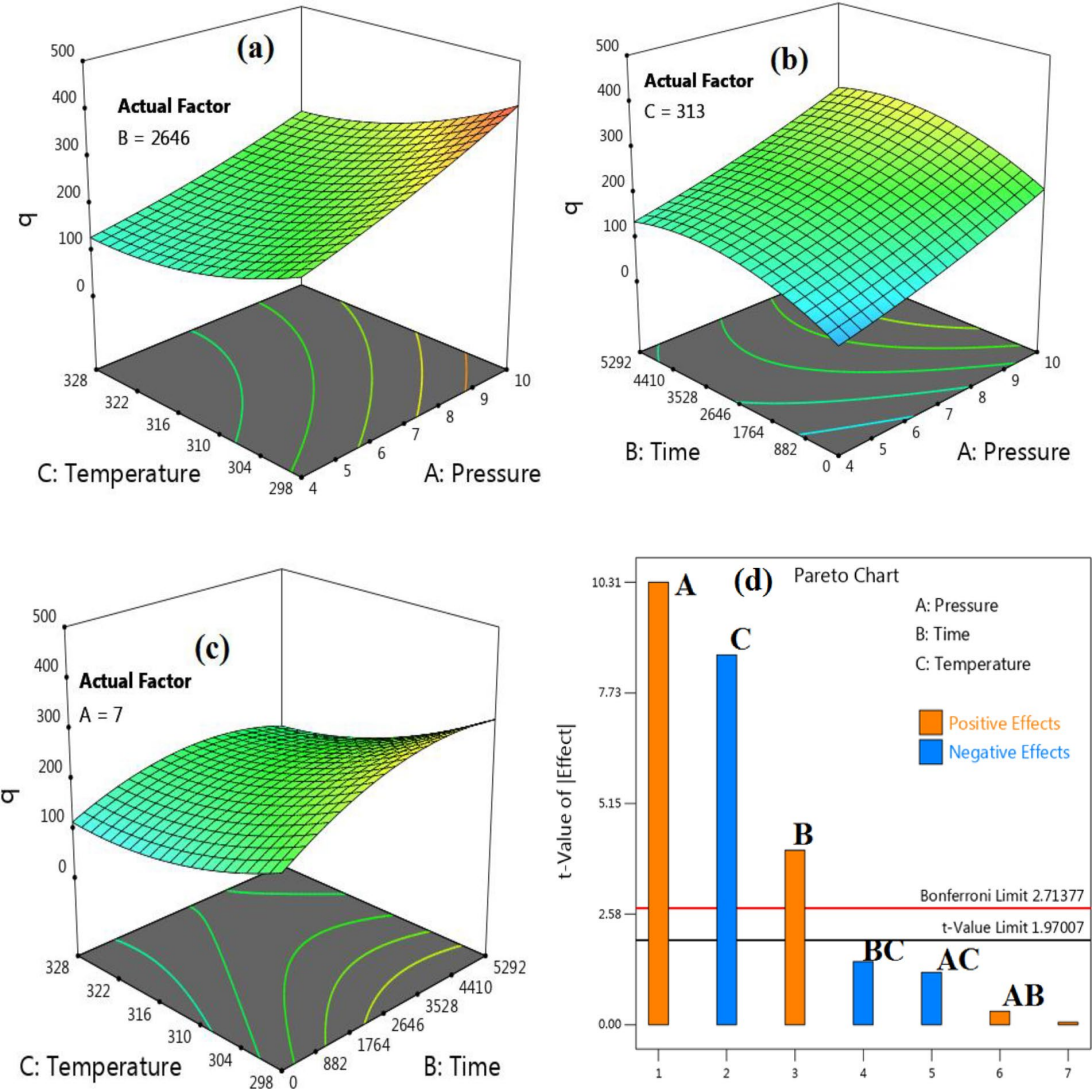
3D surface plots (**a–c**) and (**d**) Pareto chart.

To visualize the interactive effects of the variables, three-dimensional surface plots are used. These plots demonstrate how the response variable changes when one variable is varied while keeping the other variables constant. The interaction between the variables can be observed by examining the shape and pattern of the surface plot (Fig. 13a–c).

#### Pareto chart

In the realm of Response Surface Methodology (RSM), Pareto charts play a vital role in determining the most impactful factors that influence the system's response. These charts provide a visually intuitive representation of the magnitude of each factor's effect, enabling researchers to prioritize their efforts and concentrate on the variables with the greatest influence. Typically, Pareto charts in RSM arrange the factors along the x-axis in order of significance, from most to least influential^63,99–101^. The y-axis, on the other hand, represents the magnitude of the effects. The bars on the chart correspond to each factor, with the length of each bar indicating the magnitude of its effect on the response. By utilizing Pareto charts, researchers can efficiently identify the key factors that significantly impact the response, as the visual representation offers a clear understanding of their relative importance. This facilitates the identification of factors that warrant further analysis and optimization efforts. Moreover, Pareto charts in RSM can be enhanced by incorporating confidence intervals or error bars to depict the uncertainty associated with the estimated effects. This additional information aids researchers in assessing the reliability of the results and making informed decisions based on the level of confidence in the identified significant factors. In Fig. 13d, Pareto charts were utilized to visually represent the estimated effects of different parameters on CO_2_ uptake. The height of each column in the chart corresponded to the importance of the respective parameter's influence on CO_2_ uptake. Interestingly, pressure emerged as a prominent factor affecting CO_2_ uptake among the parameters examined. To assess the significance of the Pareto chart findings, an analysis of variance (ANOVA) was conducted. The results of the ANOVA provided strong evidence for the significance of the model, as indicated by a p-value less than 0.05.

### MLP-ANN

The MLP (Multi-Layer Perceptron) model is an outstanding neural network architecture that has found widespread application in various domains. Its effectiveness in learning intricate patterns and relationships within data has revolutionized problem-solving approaches. At its core, the MLP model comprises interconnected layers of artificial neurons. These neurons perform weighted summations of inputs, followed by activation functions^102^. By leveraging adjustable parameters, namely weights, and biases, the model continuously updates them during training to minimize a defined loss function^103^. This iterative process, involving forward and backward propagation, enables the MLP model to dynamically adapt and make accurate predictions or classifications on unseen data. The equations governing the behaviour of an MLP model intricately involve matrix multiplications, activation functions, and gradient calculations. These elements work together harmoniously, transforming input data into meaningful outputs. The power of the MLP model lies in its capacity to learn and generalize from large datasets, making it an invaluable tool in the realm of big data and machine learning^104^.

The MLP model calculates the weighted sum (z) of inputs for each neuron in the hidden or output layer using the formula:

17 $$ z \, = \, \sum \left( {w \, \times \, x} \right) \, + \, b $$

Here, w represents the weights of the connections, x denotes the inputs, and b signifies the bias term^66^.

#### Performance evaluation of ANN-based model

**Figure 14:**
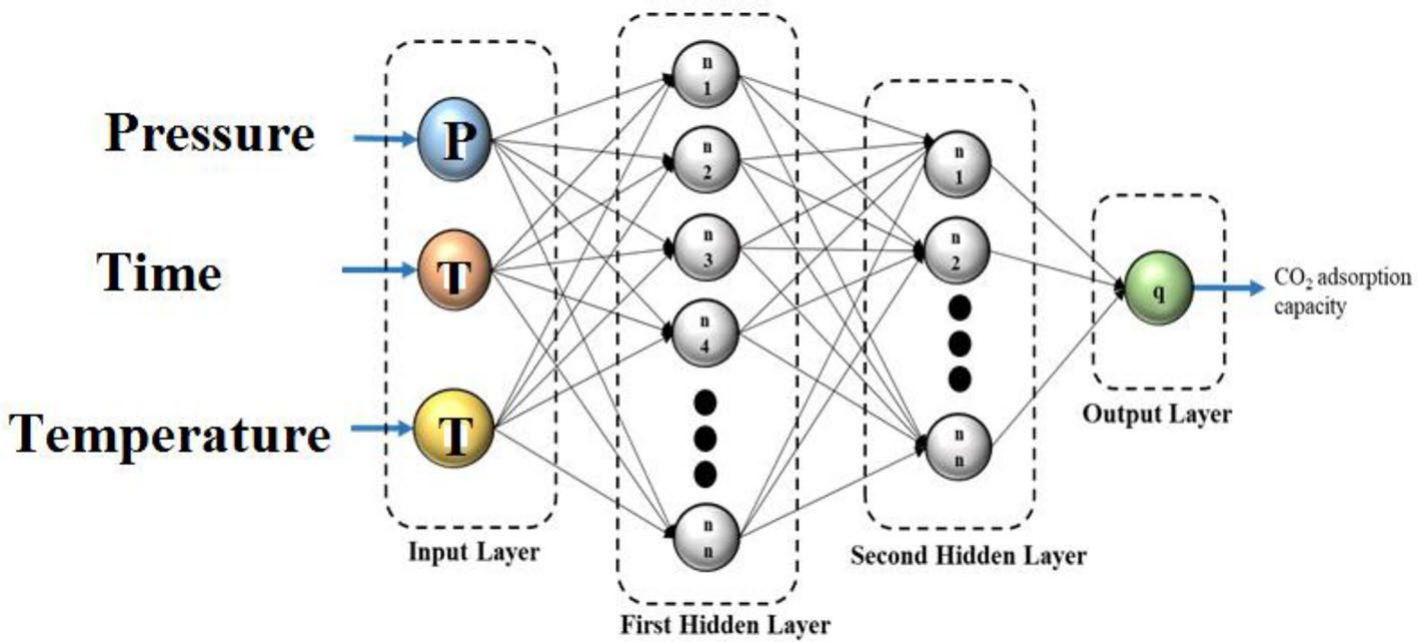
The structure of the MLP network.

The MLP model in this study is comprised of three types of layers: input layers, hidden layers, and output layers. Specifically, the MLP model used in this study consisted of four layers, which include two hidden layers, input layers, and an output layer, as depicted in Fig. 14. The ANN model was trained using the Levenberg–Marquardt backpropagation learning function, to minimize the Mean Squared Error (MSE) as the cost function. The comparison of model outputs with evaluation datasets was performed using the mean squared error (MSE) and the squared correlation coefficient (R^2^). The collected data is divided into three sets for training, testing, and validation. 70% of the data is used for training the network, 15% is used for testing the network's performance, and the remaining 15% is used for validation purposes. This division allows for evaluating the model's performance on unseen data during both the testing and validation stages.

**Figure 15:**
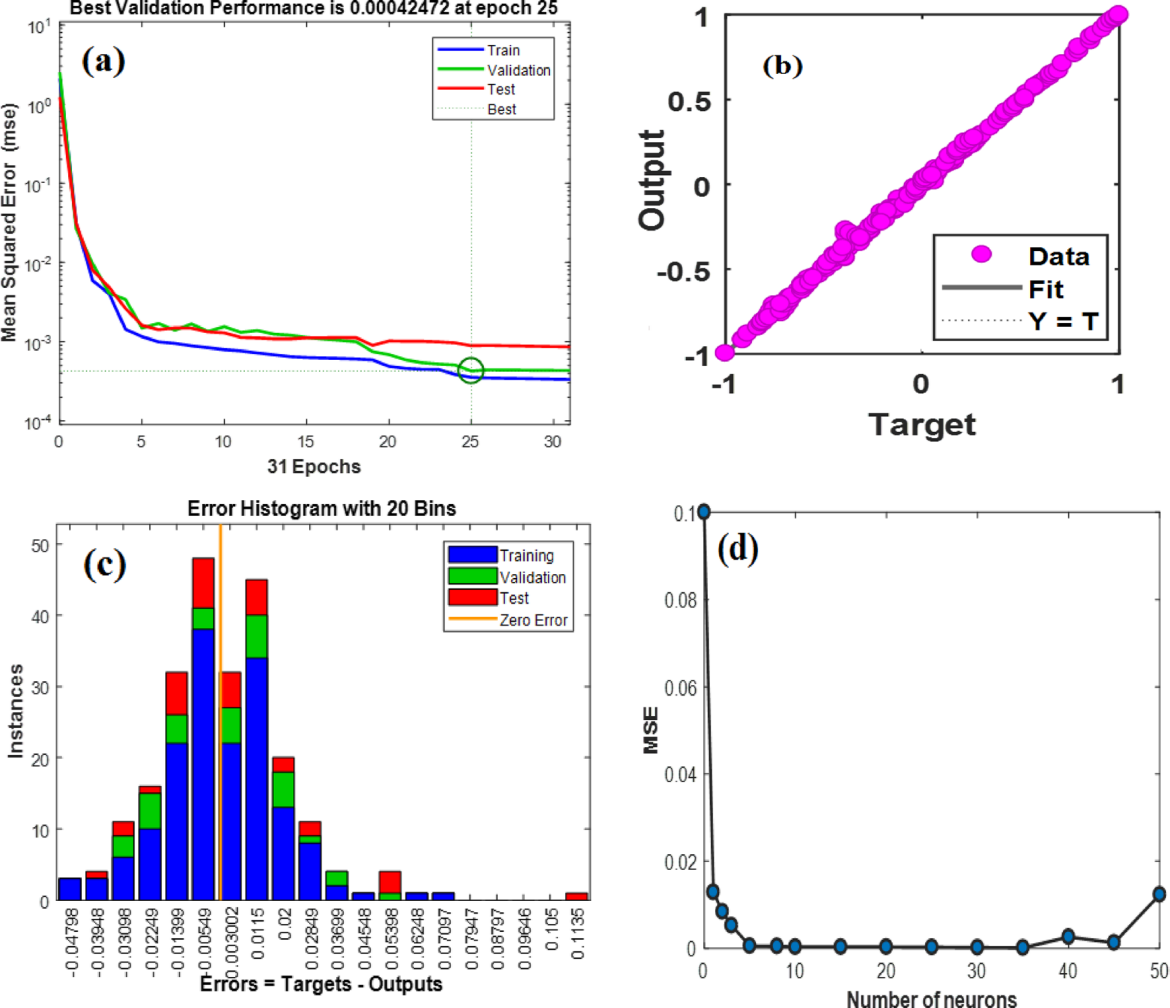
(**a**) The best validation performance of an MLP-ANN plot; (**b**) data on CO_2_ adsorption predicted versus experimented; (**c**) The error histogram plot and (**d**) the evaluation of hyperparameter.

The best validation performance of an MLP-ANN plot is an important metric that indicates the model's accuracy and generalization capabilities (Fig. 15a). In this particular case, the validation performance reached a remarkable value of 0.0004247 at epoch 25. This signifies that the MLP model achieved a high level of precision in predicting and classifying unseen data. When evaluating the performance of an MLP-ANN (Multi-Layer Perceptron Artificial Neural Network) model, one commonly used metric is R^2^, also known as the coefficient of determination. R^2^ quantifies the proportion of the variability in the predicted target variable that can be explained by the model. A high R^2^ value, such as 0.99, signifies a remarkable level of accuracy and predictive capability. With an R^2^ value of 0.99, the MLP-ANN model can account for approximately 99% of the variance in the target variable (Fig. 15b). This indicates a strong correlation between the input features and the predicted output, showcasing the model's exceptional ability to capture the underlying patterns and trends within the data.

An error histogram plot in Artificial Neural Networks (ANN) is a graphical representation that depicts the distribution of errors between the predicted and actual outputs of the model (Fig. 15c). The error histogram plot will show the frequency or count of errors at different levels of deviation from the desired output. The x-axis represents the range of error values, while the y-axis represents the frequency or count of occurrences. Since the target output in this study has a zero error of 0.0033, the histogram plot will ideally show a peak or high count at the bin corresponding to zero error. This indicates that the ANN model is performing accurately and effectively, producing outputs that closely match the desired results Overall, the error histogram plot provides valuable insights into the performance and accuracy of the ANN model. It helps in identifying patterns, trends, or any biases in the errors, enabling developers to fine-tune the model and improve its predictive capabilities. The graph in Fig. 15d illustrates the relationship between the number of neurons in the hidden layer and the minimum MSE value for this model. It is evident from the graph that as the number of neurons increases, the MSE decreases exponentially. Based on the results, the optimum neurons were selected for the hidden layer of this model. The selected MLP network structure for optimization was found to be the most suitable and efficient, comprising two hidden layers with 25 and 10 neurons, respectively.

**Figure 16:**
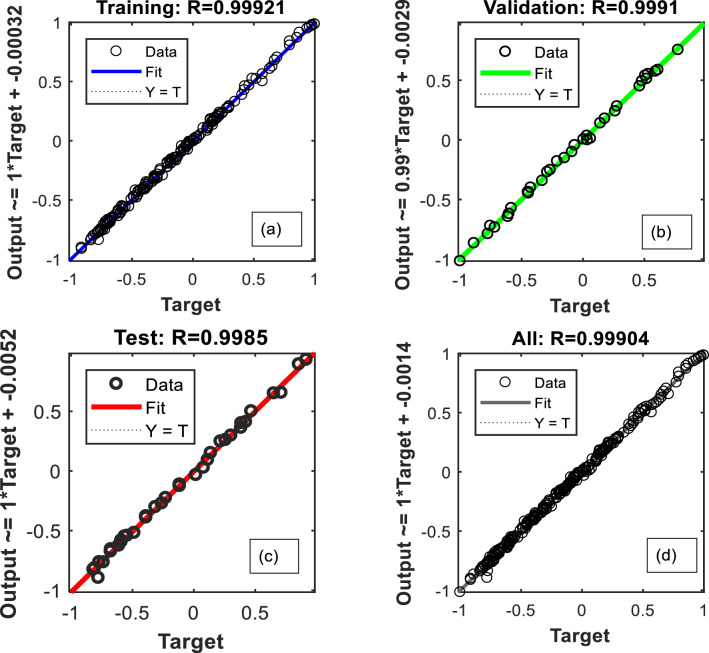
The predicted versus experimental CO_2_ adsorption using the trainlm algorithm of the MLP model. The plot includes (**a**) training, (**b**) validation, (**c**) test, and (**d**) all data.

**Figure 17:**
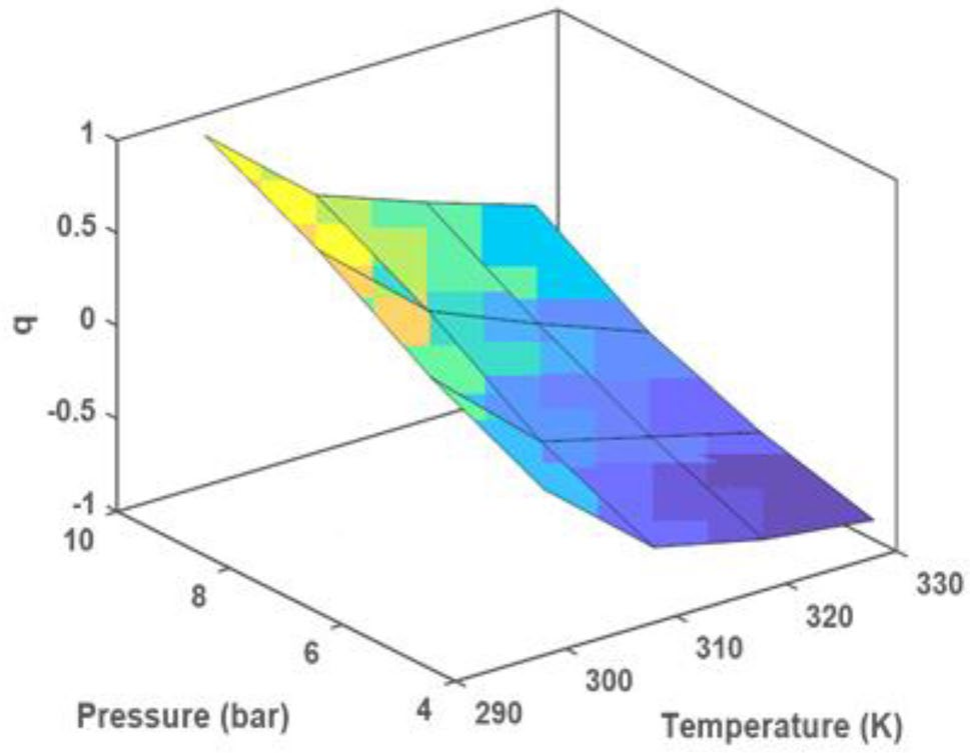
The effect of parameters on CO_2_ adsorption in ANN.

Figure 16 illustrates the comparison between predicted and experimental CO_2_ adsorption using the trainlm algorithm of the MLP (Multilayer Perceptron) model. The plot consists of four subplots:

(a) Training: This plot shows the comparison between predicted and experimental data for CO_2_ adsorption during the training phase of the MLP model. (b) Validation: This plot displays the predicted versus experimental CO_2_ adsorption during the validation phase of the MLP model. (c) Test: The predicted and experimental CO_2_ adsorption data are compared in this subplot for the test phase of the MLP model. (d) All data: This plot combines all the data points, including training, validation, and test data, to provide an overall comparison between predicted and experimental CO_2_ adsorption. This plot helps in evaluating the performance of the MLP model by visually assessing the agreement between predicted and experimental CO_2_ adsorption under different data subsets. Figure 17 illustrates the impact of parameters on CO_2_ adsorption through 3D plots in ANN.

### Compression of RSM and ANN models

**Figure 18:**
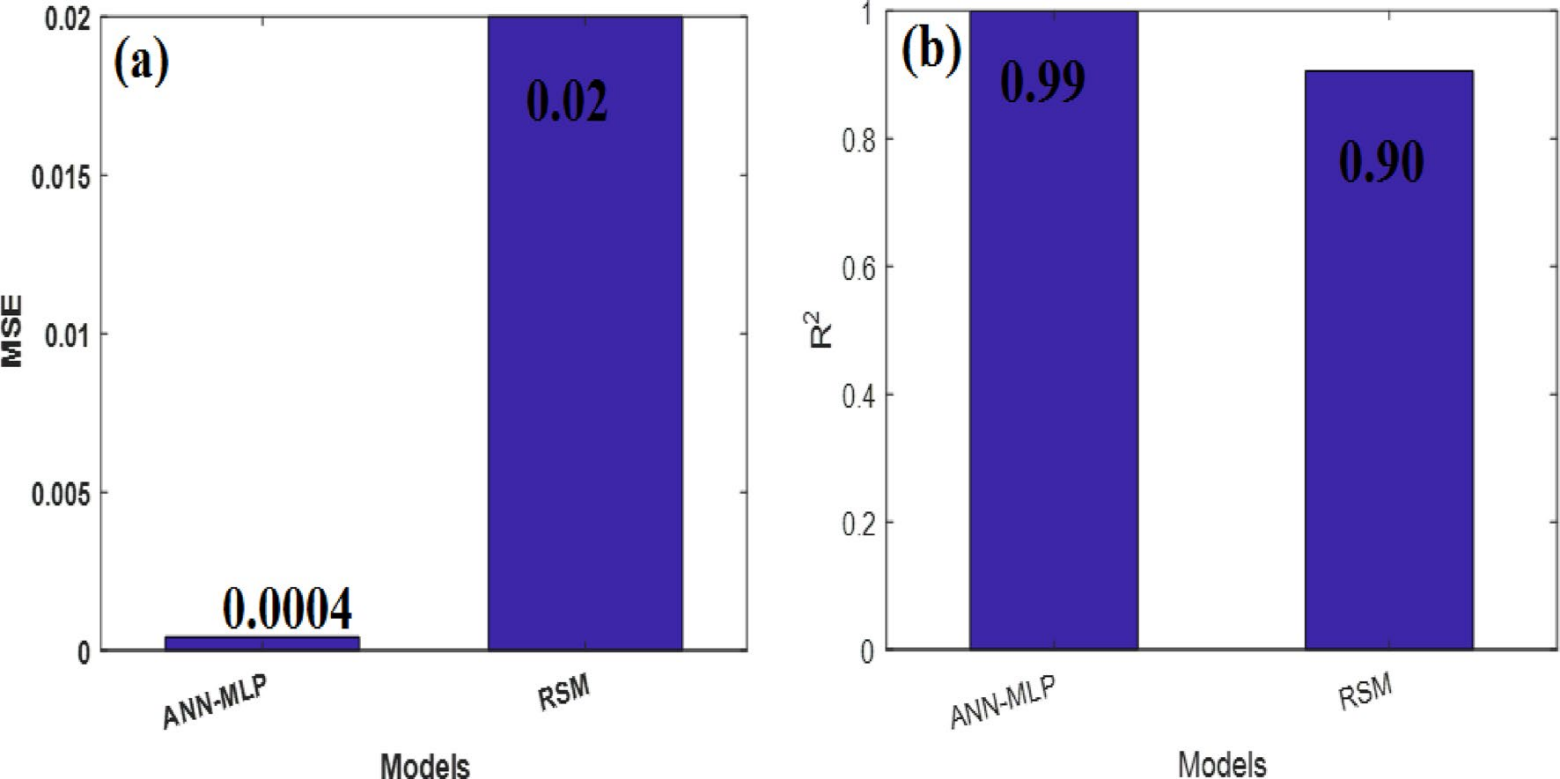
The comparison of ANN and RSM models based on (**a**) MSE and (**b**) R^2^.

The present study compared the efficacy of two models, namely the RSM (Response Surface Methodology) model and the ANN (Artificial Neural Network) model, in the context of compression. To assess the performance of each model, two evaluation metrics were employed: mean squared error (MSE) and R-squared (R^2^). MSE, a metric that gauges the average squared difference between the predicted and actual values, serves as an indicator of overall model accuracy. Lower MSE values are indicative of superior performance. Conversely, R^2^ measures the extent to which the independent variables explain the variance in the dependent variable. Higher R^2^ values suggest a more optimal fit of the model to the data. The coefficient of determination (R^2^) of RSM was reported to be 0.90. The MSE was found to be 0.02. Figure 18 illustrates the comparison between the RSM and ANN models in terms of their performance in compression. This indicates a strong level of predictive power and a good fit of the model to the data. Upon careful examination of the results, it was observed that the MLP (Multi-Layer Perceptron) model exhibited the lowest MSE (0.0004) and a considerably high R^2^ value (0.99). This finding implies that the MLP model could predict compressed data with minimal error and effectively explain the variability in the dependent variable. Based on these compelling outcomes, it was concluded that the MLP model surpassed the RSM model.

### The Pearson correlation matrix

**Figure 19:**
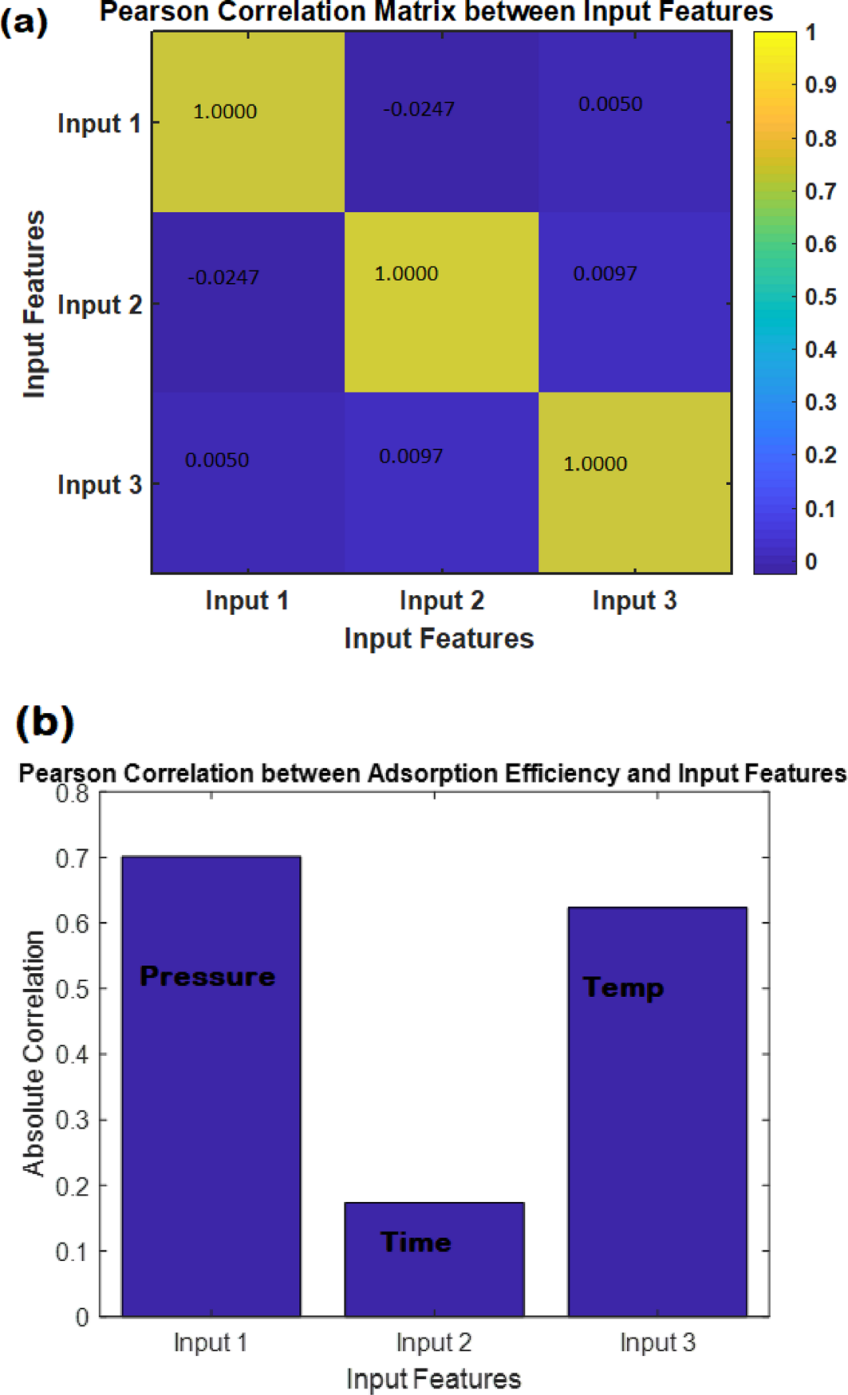
(**a**) Pearson correlation matrix and (**b**) Pearson correlation bar plot.

The Pearson correlation matrix is a square matrix that displays the correlation coefficients among pairs of input features within a dataset (Fig. 19). These coefficients quantify the degree of linear relationship between variables and range from − 1 to + 1. A correlation of + 1 denotes a perfect positive correlation, − 1 signifies a perfect negative correlation, and 0 indicates no linear correlation. The diagonal elements always have a correlation coefficient of 1, representing a variable's correlation with itself. Analyzing the correlation matrix provides valuable insights into the dataset. The magnitude and sign of correlation coefficients reveal the strength and direction of relationships between variables. For instance, a coefficient near + 1 suggests a robust positive correlation, indicating that an increase in one variable corresponds to an increase in the other. Conversely, a coefficient close to − 1 implies a strong negative correlation, suggesting an inverse relationship.

Examining the Pearson correlation coefficient matrix reveals that the covariance between Pressure and CO_2_ uptake capability is directly proportional to the product of their standard deviations^105,106^. Specifically, the correlation coefficient between Pressure and CO_2_ uptake capability is 1, indicating a strong positive relationship. This signifies that as Pressure increases, the CO_2_ uptake capability also increases proportionally.

### The regeneration of adsorbents

**Figure 20:**
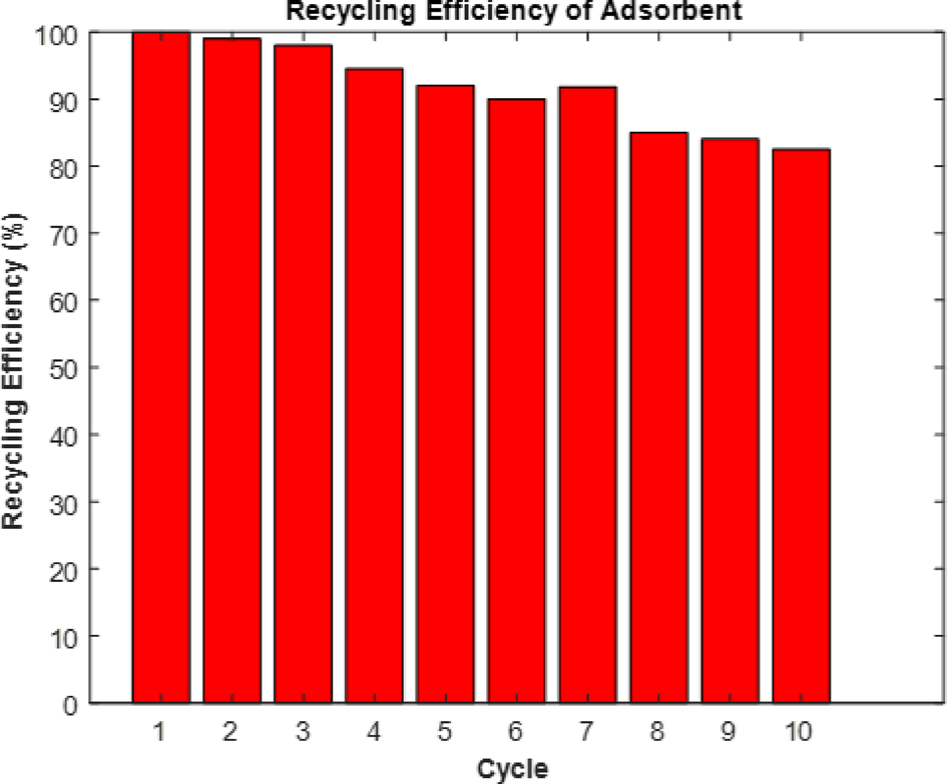
Cycling efficiency of M-MWCNT for carbon dioxide capture.

The regeneration of adsorbents plays a crucial role in the process of CO_2_ capture, as it allows for the reuse of the adsorbent material and the release of captured CO_2_^107,108^. In the context of CO_2_ capture, a unique and effective regeneration process involves multiple cycles of adsorption and desorption, typically around 10 cycles. During the adsorption phase, the adsorbent material, which is often a porous solid selectively captures CO_2_ from a gas stream. The adsorption process is driven by the affinity of the adsorbent material towards CO_2_, allowing it to selectively adsorb CO_2_ molecules while allowing other gases to pass through. After the adsorption phase, the adsorbent material becomes saturated with CO_2_ and needs to be regenerated to release the captured CO_2_. The regeneration process involves subjecting the adsorbent material to elevated temperatures, typically in the range of 100–200 °C, and reducing the pressure to release the adsorbed CO_2_. This desorption process allows the adsorbent material to be regenerated and ready for the next cycle of CO_2_ capture. The regeneration process of solid adsorbents in the TSA (temperature swing adsorption) plays a crucial role in enhancing their lifetime and reducing the relative cost of CO_2_ adsorption^109,110^. In this process, the adsorbent used for CO_2_ adsorption is cyclically consumed and regenerated through multiple adsorption and to regenerate the adsorbent, it is first dried in a vacuum oven at 100 °C for 8 h after each adsorption cycle. This prepares it for the subsequent adsorption cycles. The regenerated adsorbent can be reused multiple times for the CO_2_ adsorption process, as demonstrated in the study. Specifically, the adsorbent was reused 10 times at 25 °C and 8 bar for CO_2_ adsorption. However, it is worth noting that the adsorption capacity of M-MWCNT adsorbent decreased from 100 to 82% after 10 cycles, as shown in Fig. 20. Despite the decrease in adsorption capacity, the results of the regeneration process indicate that the M-MWCNT adsorbent can still be employed on due high-value adsorption capacity. In summary, the regeneration process discussed allows for the repeated use of the adsorbent, thereby extending its lifetime and reducing the overall cost of CO_2_ adsorption. The findings suggest that the M-MWCNT adsorbent has the potential for industrial-scale applications due to its favourable adsorption capacity.

### Comparison studies

A comparison study was conducted to evaluate the CO_2_ adsorption capacity of multi-walled carbon nanotubes (MWCNTs) in Table 10. In this study, MWCNTs were synthesized via CVD using a Fe–Ni-activated carbon catalyst.

**Table 10:** A comparison study to evaluate the CO_2_ adsorption capacity of MWCNTs.

Adsorbents/methods	T(C)	CO_2_ sorption capacity (mg/g)	References
MWCNT modified with Monoethanolamine	25	26.9	[91]
MWCNT modified with Polyethylenimine	20	30	[92]
MWCNT modified with 1,3-diaminopropane	30	92.71	[93]
MWCNT modified with PEI (+ silica coating)	25	62.17	[94]
MWCNT modified with Fe_2_O_3_/Al_2_O_3_ composite by CVD methods	25	129	[34]
MWCNT: Fe–Ni/AC catalyst by CVD methods	25	421.08	This work

## Conclusion

This study successfully produced modified multi-walled carbon nanotubes (MWCNTs) through chemical vapour deposition (CVD) to enhance CO_2_ adsorption. The modified MWCNTs exhibited a reduced surface area but demonstrated a significant increase in adsorption capacity, especially at higher pressure and lower temperature conditions. The reduction in surface area of multi-walled carbon nanotubes (MWCNTs) from 240 to 11 m^2^/g after modification can be attributed to several factors. Modification of MWCNTs involves the functionalization or introduction of additional groups or molecules on the surface of the nanotubes. This can lead to various effects, including the blocking of existing surface sites and the creation of new surface sites, which results in a decrease in available surface area.

However, despite the decrease in surface area, the modified MWCNTs show an increased adsorption capacity for CO_2_ at higher pressure and lower temperatures. This phenomenon can be understood through the principles of adsorption kinetics and thermodynamics. At higher pressures, more CO_2_ molecules are available for adsorption, and the modified MWCNTs can accommodate a larger number of these molecules. This can be attributed to the creation of additional adsorption sites or the enhancement of existing ones through the modification process. The functionalization of MWCNTs can introduce chemical groups that have a higher affinity for CO_2_, leading to increased adsorption capacity. At lower temperatures, the adsorption process is favoured thermodynamically. When the temperature decreases, the thermal energy of CO_2_ decreases, making it more prone to adsorption on the modified MWCNTs. Additionally, the modification process might result in interactions between CO_2_ molecules and the functional groups on the surface of MWCNTs that are more favourable at lower temperatures. The adsorption process was found to be exothermic and spontaneous. A feed-forward MLP artificial neural network model, optimized with two hidden layers and trained using the Levenberg–Marquardt backpropagation algorithm, accurately predicted CO_2_ adsorption. The study also employed response surface methodology (RSM) to further enhance adsorption prediction. A noteworthy observation from this study is that the adsorption capacity of the modified multi-walled carbon nanotube (M-MWCNT) adsorbent decreased from 100 to 82% after undergoing 10 cycles. However, even with this decrease, the regeneration process showed promising results, indicating that the M-MWCNT adsorbent can still be effectively utilized due to its high-value adsorption capacity. These findings highlight the potential of modified MWCNTs for efficient gas adsorption in various industrial applications. The MLP model demonstrated superior performance with the lowest MSE (0.0004) and a high R^2^ value (0.99), indicating its accurate prediction capabilities and strong explanatory power compared to the RSM model.

## Data Availability

The datasets used and analyzed during the current study are available from the corresponding author upon reasonable request.
